# Femoral specializations to locomotor habits in early archosauriforms

**DOI:** 10.1111/joa.13598

**Published:** 2021-11-28

**Authors:** Romain Pintore, Alexandra Houssaye, Sterling J. Nesbitt, John R. Hutchinson

**Affiliations:** ^1^ Structure and Motion Laboratory Department of Comparative Biomedical Sciences Royal Veterinary College Hatfield UK; ^2^ Mécanismes adaptatifs et évolution (MECADEV)/UMR 7179 CNRS/Muséum National d’Histoire Naturelle Paris France; ^3^ Department of Geosciences Virginia Tech Blacksburg Virginia USA

**Keywords:** appendicular skeleton, Archosauria, body size, functional morphology, geometric morphometrics, locomotion, Triassic

## Abstract

The evolutionary history of archosaurs and their closest relatives is characterized by a wide diversity of locomotor modes, which has even been suggested as a pivotal aspect underlying the evolutionary success of dinosaurs vs. pseudosuchians across the Triassic–Jurassic transition. This locomotor diversity (e.g., more sprawling/erect; crouched/upright; quadrupedal/bipedal) led to several morphofunctional specializations of archosauriform limb bones that have been studied qualitatively as well as quantitatively through various linear morphometric studies. However, differences in locomotor habits have never been studied across the Triassic–Jurassic transition using 3D geometric morphometrics, which can relate how morphological features vary according to biological factors such as locomotor habit and body mass. Herein, we investigate morphological variation across a dataset of 72 femora from 36 different species of archosauriforms. First, we identify femoral head rotation, distal slope of the fourth trochanter, femoral curvature, and the angle between the lateral condyle and crista tibiofibularis as the main features varying between bipedal and quadrupedal taxa, all of these traits having a stronger locomotor signal than the lesser trochanter's proximal extent. We show a significant association between locomotor mode and phylogeny, but with the locomotor signal being stronger than the phylogenetic signal. This enables us to predict locomotor modes of some of the more ambiguous early archosauriforms without relying on the relationships between hindlimb and forelimb linear bone dimensions as in prior studies. Second, we highlight that the most important morphological variation is linked to the increase of body size, which impacts the width of the epiphyses and the roundness and proximodistal position of the fourth trochanter. Furthermore, we show that bipedal and quadrupedal archosauriforms have different allometric trajectories along the morphological variation in relation to body size. Finally, we demonstrate a covariation between locomotor mode and body size, with variations in femoral bowing (anteroposterior curvature) being more distinct among robust femora than gracile ones. We also identify a decoupling in fourth trochanter variation between locomotor mode (symmetrical to semi‐pendant) and body size (sharp to rounded). Our results indicate a similar level of morphological disparity linked to a clear convergence in femoral robusticity between the two clades of archosauriforms (Pseudosuchia and Avemetatarsalia), emphasizing the importance of accounting for body size when studying their evolutionary history, as well as when studying the functional morphology of appendicular features. Determining how early archosauriform skeletal features were impacted by locomotor habits and body size also enables us to discuss the potential homoplasy of some phylogenetic characters used previously in cladistic analyses as well as when bipedalism evolved in the avemetatarsalian lineage. This study illuminates how the evolution of femoral morphology in early archosauriforms was functionally constrained by locomotor habit and body size, which should aid ongoing discussions about the early evolution of dinosaurs and the nature of their evolutionary “success” over pseudosuchians.

## Graphical abstract

We studied femoral shape variation by applying 3D geometric morphometrics to a large sample of archosauriforms predominantly from the Late Triassic. We identified a set of anatomical features varying with locomotor mode (quadrupedal/bipedal) or body size. We also showed that morphological variation linked to locomotor mode had a strong phylogenetic signal and that femoral shape specialization to body size was convergent between avemetatarsalians (=dinosaur lineage) and pseudosuchians (=crocodile lineage).

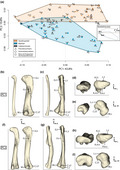

## 1 INTRODUCTION

Archosauriformes is a saurian clade that largely radiated in the Triassic Period, and an exceptional diversity of locomotor habits is one of its most striking features (Bakker, 1971; Bates & Schachner, 2012; Bishop et al., 2020; Bonaparte, 1984; Chapelle et al., 2020; Charig, 1972; Demuth et al., 2020; Hutchinson, 2006; Hutchinson & Gatesy, 2000; Parrish, 1986; Sereno, 1991; Tsai et al., 2019). In addition to the distinct lifestyles and locomotor habits of the crown groups Aves and Crocodylia, accounting for extinct archosaurs over the last 250 million years broadens this scope dramatically, with animals that were terrestrial, semi‐aquatic, or volant; with more sprawling to more erect limb postures, or with bipedal to quadrupedal locomotor modes (Carrano, 1999; Grinham et al., 2019; Kubo & Kubo, 2012; Otero et al., 2019; Persons & Currie, 2017). Additionally, archosauriforms show great variation in body size, with animals ranging from very small to the largest land vertebrates that have ever existed (e.g., a few centimetres to tens of metres in total length, Benson et al., 2017; Campione & Evans, 2020; Colbert, 1962; Kammerer et al., 2020; Tsai et al., 2020). This unique variation of biological parameters inevitably impacted the skeleton of archosauriforms, especially limb bones given that they are subjected to biomechanical constraints due to body support requirements and locomotor habits (Bakker, 1971; Carrano, 1998; Charig, 1972; Coombs, 1978; Hutchinson, 2006; Hutchinson & Gatesy, 2000; Parrish, 1986; Tsai et al., 2020). These skeletal specializations have been suggested to help explain the evolutionary success of dinosaurs vs. pseudosuchians across the Triassic–Jurassic transition (Bakker & Galton, 1974; Bonaparte, 1984; Parrish, 1986). Archosaurian success itself might have been facilitated by the acquisition of an erect limb posture, which led to the origin of obligate bipedalism in some lineages in the Triassic. These changes appeared correlated with a lower disparity (morphological diversity) in small, putatively more athletic avemetatarsalians than in the well‐established pseudosuchians at the end of the Late Triassic (Bakker & Galton, 1974; Charig, 1972; Irmis, 2011; Kubo & Kubo, 2012; Sullivan, 2015). However, this hypothesis of morphofunctional disparity is challenged by the discovery of new species reducing the gap of disparity between pseudosuchians and avemetatarsalians at the end of the Late Triassic, as well as increasing the amount of functional and morphological convergences between Avemetatarsalia and Pseudosuchia in the last four decades (Bates & Schachner, 2012; Brusatte et al., 2008a, 2008b; Carrano, 2000; Foth et al., 2016, 2021; Gatesy, 1991; Grinham, 2019; Nesbitt & Norell, 2006; Novas et al., 2021; Parrish, 1986, 1987; Singh et al., 2021; Toljagić & Butler, 2013). Indeed, discoveries of small bipedal pseudosuchians such as *Shuvosaurus* (Chatterjee, 1993; Nesbitt & Norell, 2006) and the larger *Poposaurus* (Gauthier et al., 2011), and the possibility that larger pseudosuchians like *Postosuchus* (Weinbaum, 2013) and *Riojasuchus* (Walker, 1964; Baczko et al., 2020) were facultative or obligatory bipeds, blur the line of morphofunctional distinction between the two clades. The same phenomenon applies to early avemetatarsalians, with the recently discovered early diverging taxon *Teleocrater* (Nesbitt et al., 2017) being quadrupedal, and some early dinosauriforms such as *Silesaurus* and *Asilisaurus* (Nesbitt et al., 2010; Piechowski & Dzik, 2010) perhaps being obligate quadrupeds or only facultatively bipedal. Kubo and Kubo (2012) showed that the multiple origins of bipedalism among early archosauriforms seemed correlated with a “cursoriality index,” corresponding to the metatarsal III vs. femur relative lengths. This cursoriality index was higher in bipedal avemetatarsalians than in bipedal pseudosuchians and was suggested to be a key factor in the dinosaur radiation and the extinction of most Triassic pseudosuchians. Additionally, Kubo and Kubo (2012) found that body size was negatively correlated with the origin of cursorial morphology among ornithodirans but not among pseudosuchians, suggesting a potentially important relationship between locomotor mode, limb posture, and body size with morphological variation of the femur and metatarsus. If correct, these ideas of Kubo and Kubo (2012; also see Kubo & Kubo, 2013, 2016) might help explain why some avemetatarsalians fared better than most pseudosuchians during the environmental upheavals around the Triassic–Jurassic boundary, but do not show a clear explanatory relationship with faunal changes earlier in the Triassic (e.g. Irmis, 2011; Novas et al., 2021).

There has been extensive debate over which factors may have led to the rise and early success of archosaurs in the early Mesozoic (Irmis, 2011). Previous hypotheses have centered on limb posture in amniotes, namely that erect (adducted) limb posture favored archosaurs over synapsids, or dinosaurs over pseudosuchians (e.g., Bakker & Galton, 1974; Bonaparte, 1984; Charig, 1972; Parrish, 1986). These hypotheses have essentially been refuted, but explanations for differential survival of amniotes through the Triassic and taxa across the Triassic–Jurassic boundary remain contentious (e.g., Benton, 1983; Brusatte et al., 2008a, 2008b; Irmis, 2011; Kubo & Kubo, 2012; Toljagić & Butler, 2013; Foth et al., 2016, 2021; Singh et al., 2021). Disparity analyses have featured prominently more recently. In these analyses, taxon sampling has been increased for pseudosuchians and non‐archosaurian Archosauriformes and disparity has been measured using isolated skeletal regions (e.g., crania, pelvic, and/or limb elements exclusively) or entire skeletons by means of phylogenetic characters (Foth et al., 2016, 2021; Kubo & Kubo, 2012; Stubbs et al., 2013; Toljagić & Butler, 2013). However, the functional morphology and disparity of early archosauriforms’ limb bones has never been investigated using quantitative analyses on a large and representative sample of the same skeletal element and remains relevant to ongoing debates.

Here, we quantify the similarities and differences between early archosauriform femora accounting for their locomotor habit and body size using three‐dimensional geometric morphometrics (3D GMM). We investigate which femoral features have a strong phylogenetic signal, and how they relate to the divergence between stem crocodylians (=pseudosuchians) and stem avians (=avemetatarsalians). We also investigate convergence in femoral shape among archosauriforms and how it relates to functional factors such as body size and locomotor mode. 3D GMM is well suited to morphofunctional studies at the level of the appendicular skeleton, even for extinct species (Hedrick et al., 2020; Lefebvre et al., 2020; Maclaren et al., 2018; Martin‐Serra et al., 2014; Milne et al., 2009; Paramo et al., 2020). In addition, 3D GMM has the potential to give new insights into femoral morphological variation in archosauriforms, which seems to be strongly three‐dimensional (e.g., Parrish, 1986), whereas the rodlike metatarsals seem to vary mainly in their relative length and in the number of constituent bones. By applying 3D GMM, we can test whether the apparent differences of locomotor modes relate to specific features such as femoral head rotation and anterior bowing of the femur, and address how any differences relate to the continuum between graviportal and cursorial (*sensu* Carrano, 1999) morphologies; including traits such as femoral robusticity and position of the fourth trochanter. 3D GMM offers a unique opportunity to identify morphological features associated with locomotor mode and body size as well as their covariation, ultimately deepening our understanding of the morphofunctional basis of locomotion in the evolutionary history of early archosauriforms. This understanding can in turn feed into future work re‐examining the reasons for archosauriforms and avemetatarsalians “success” during the Triassic.

## 2 MATERIALS AND METHODS

### 2.1 Sample

**FIGURE 1:**
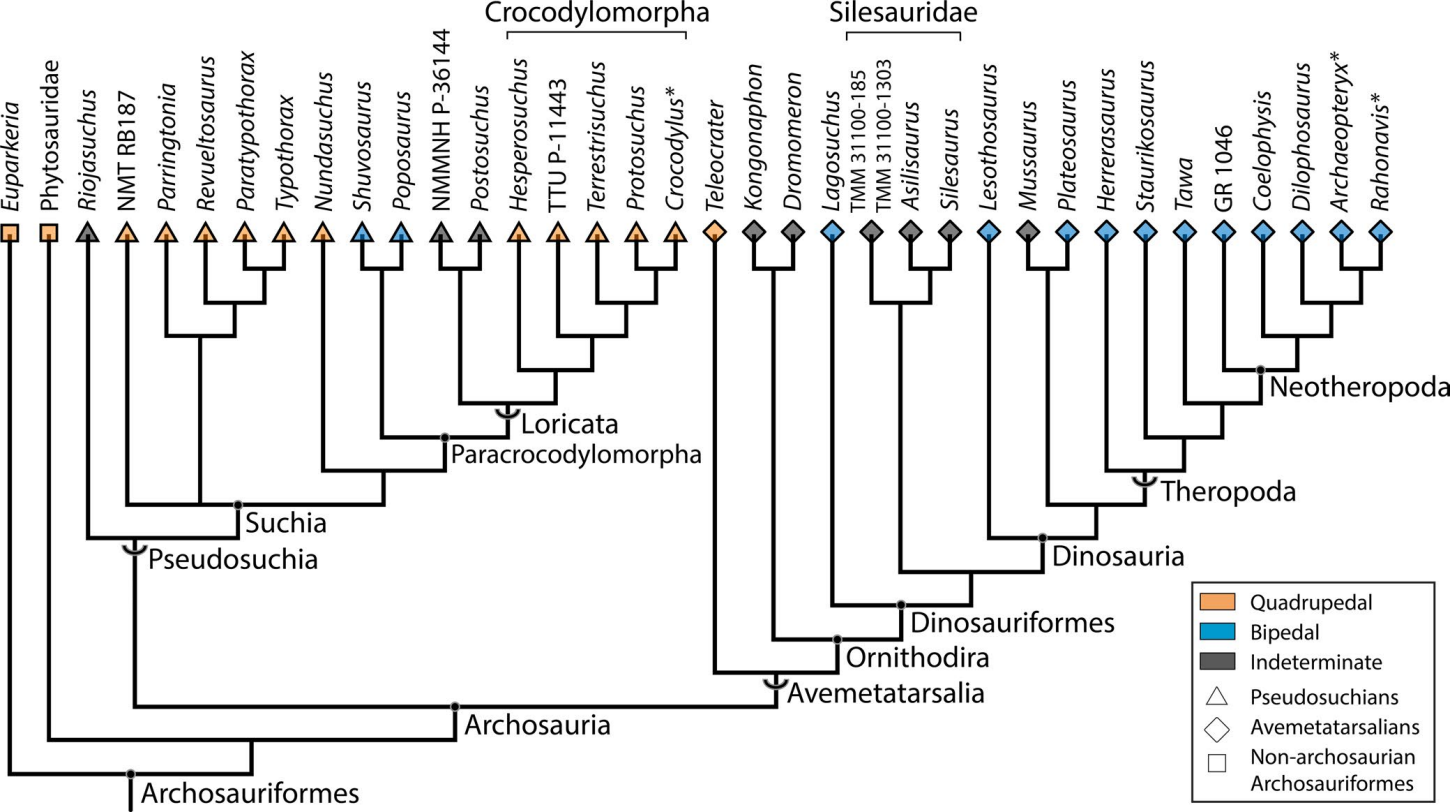
Phylogenetic tree of studied Archosauriformes based on Nesbitt (2011), Nesbitt et al. (2014) and Butler et al. (2017). Clade type shown with: chevron, stem‐based clades; node, node‐based clades. Bracketing taxa that lived well after the Triassic–Jurassic transition are highlighted with a * after their genus name

Our sample comprised 72 femora from 36 species of archosauriforms, including 32 femora from 16 species of pseudosuchians, 37 femora from 18 species of avemetatarsalians, and 3 femora from 2 species of non‐archosaurian archosauriforms (Table 1, Figure 1). Specimens were selected in order to best represent the disparity of early archosauriform limb bones between the Late Triassic and the Early Jurassic, within the constraint of availability and suitability for 3D digitization or CT scanning (including quality of 3D taphonomic preservation, especially of the epiphyses; see below). *Euparkeria* is often placed phylogenetically as one of the closest outgroups to crown Archosauria (Ezcurra et al., 2020; Nesbitt, 2011; Sookias, 2016) and was thus integrated into the sample, along with the two phytosaurs, which may or may not be crown archosaurs (Brusatte et al., 2010; Ezcurra, 2016; Nesbitt, 2011). The Triassic pseudosuchians in our sample were represented by the armoured aetosaurs and their sister‐taxon *Revueltosaurus* (Nesbitt, 2011; Parker, 2016), ornithosuchids, poposauroids, loricatans (“rauisuchians” and related taxa *sensu* Nesbitt, 2011) as well as crocodylomorphs (Table 1). The Triassic and Early Jurassic avemetatarsalians in our sample were represented by aphanosaurians (*Teleocrater*), lagerpetids (*Kongonaphon*, *Dromomeron*), the earliest diverging dinosauriform *Lagosuchus*, silesaurids, early Ornithischia (*Lesothosaurus*), and possible early theropods including *Herrerasaurus* and *Staurikosaurus* along with Late Triassic and Early Jurassic sauropodomorphs and theropods (Table 1). In addition, taxa without any major ambiguities regarding their locomotor mode (see Grinham et al., 2019) were used as bracketing taxa for bipedal and quadrupedal morphologies in order to polarize the main variation. Subadult and juvenile extant Nile crocodiles (*Crocodylus niloticus*) were selected to represent quadrupedal archosaurs. Bipedal archosaurs could not be represented by living taxa—avian dinosaurs—because of particular anatomical fusions (e.g., greater and lesser trochanters fused into one trochanteric crest [Carrano, 2000; Hutchinson, 2001]) that rendered it impossible to digitize homologous landmarks in the right orientation and along the correct structures (Gunz & Mitteroecker, 2013). Thus, unambiguously bipedal theropods closely related to avian theropods that lived after the Triassic to Jurassic transition were selected: the avialan *Archaeopteryx* from the Late Jurassic and *Rahonavis* from the Late Cretaceous. When available, left and right femora from the same fossil specimen were used (three out of 69 individuals).

**TABLE 1:** List of all femora included in this study

Higher order	Species	Abb.	Institution	Nb.	Loc.	Side(s)	FL	Dig.
Non‐archosaurian Archosauriformes	*Euparkeria capensis*	Eup	SAM PK	5867	Q	R	52.2	µCT
Non‐archosaurian Archosauriformes	Phytosauridae	Phy	PEFO	23347	Q	L	375.4	Ph
Non‐archosaurian Archosauriformes	Phytosauridae	Phy	PEFO	31219	Q	L	413.3	Ph
Pseudosuchia, Erpetosuchidae	*Parringtonia gracilis*	Par	NMT	RB188	Q	R	71.9	SS
Pseudosuchia, Erpetosuchidae	*Parringtonia gracilis*	Par	NMT	RB246	Q	L, R	70.6, 70.2	SS
Pseudosuchia, Ornithosuchidae	*Riojasuchus tenuisceps*	Rio	PVL	3827	C	L	154.4	CT
Pseudosuchia, Ornithosuchida	*Riojasuchus tenuisceps*	Rio	PVL	3828	C	L	170.7	CT
Pseudosuchia, Suchia	Suchia indet.	Suc	NMT	RB187	Q	R	138.7	SS
Pseudosuchia, Suchia	*Revueltosaurus callenderi*	Rev	PEFO	34269	Q	L	81.5	Ph
Pseudosuchia, Suchia	*Revueltosaurus callenderi*	Rev	PEFO	34561	Q	L	92.1	Ph
Pseudosuchia, Aetosauridae	*Paratypothorax* sp.	Par	TTUP	12547	Q	R	480.5	Ph
Pseudosuchia, Aetosauria	*Typothorax coccinarum*	Typ	NMMNH	P‐11775	Q	L	198	Ph
Pseudosuchia, Aetosauria	*Typothorax coccinarum*	Typ	NMMNH	P‐11778	Q	L	254.9	Ph
Pseudosuchia, Paracrocodylomorpha	*Nundasuchus songeaensis*	Nun	NMT	RB48	Q	R	230.9	SS
Pseudosuchia, Poposauridea	*Poposaurus gracilis*	Pop	YPM	57100	B	L, R	333.8, 339.9	CT
Pseudosuchia, Poposauridea	*Shuvosaurus inexpectatus*	Shu	NMMNH	P‐4695	B	R	128.7	Ph
Pseudosuchia, Poposauridea	*Shuvosaurus inexpectatus*	Shu	TTUP	18307	B	L	202.7	Ph
Pseudosuchia, Poposauridea	*Shuvosaurus inexpectatus*	Shu	TTUP	18321	B	L	197.6	Ph
Pseudosuchia, Poposauridea	*Shuvosaurus inexpectatus*	Shu	TTUP	18336	B	L	241.1	Ph
Pseudosuchia, Poposauridea	*Shuvosaurus inexpectatus*	Shu	TTUP	9001	B	L	230.9	SS
Pseudosuchia, Loricata	Loricata indet.	Lor	NMMNH	P‐36144	C	R	374.8	Ph
Pseudosuchia, Rauisuchidae	*Postosuchus kirkpatricki*	Pos	TTUP	9000	C	L	504.5	Ph
Pseudosuchia, Rauisuchidae	*Postosuchus kirkpatricki*	Pos	TTUP	9002	C	L, R	373.7, 388.3	Ph
Pseudosuchia, Crocodylomorpha	*Hesperosuchus agilis*	Hes	AMNH	FR6758	Q	L	136.5	SS
Pseudosuchia, Crocodylomorpha	Crocodylomorpha indet.	Crm	TTUP	11443	Q	R	109.8	Ph
Pseudosuchia, Crocodylomorpha	*Terrestrisuchus gracilis*	Ter	NHMUK PV	R7562	Q	R	82.1	µCT
Pseudosuchia, Crocodylomorpha	*Terrestrisuchus gracilis*	Ter	NHMUK PV	R10002	Q	R	63.7	µCT
Pseudosuchia, Crocodylomorpha	*Terrestrisuchus gracilis*	Ter	Composite of proximal R7562 and distal R10002	NA	Q	R	54.4	µCT
Pseudosuchia, Crocodylomorpha	*Protosuchus richardsoni*	Pro	AMNH	3024	Q	R	110.2	CT
Pseudosuchia, Crocodylomorpha	Crocodylus niloticus*	Cro	RVC	DDNC01	Q	R	66.8	CT
Pseudosuchia, Crocodylomorpha	Crocodylus niloticus*	Cro	RVC	DDNC02	Q	R	49.2	CT
Pseudosuchia, Crocodylomorpha	Crocodylus niloticus*	Cro	RVC	DDNC03	Q	R	58.9	CT
Pseudosuchia, Crocodylomorpha	Crocodylus niloticus*	Cro	RVC	DDNC04	Q	R	70.5	CT
Pseudosuchia, Crocodylomorpha	*Crocodylus niloticus*	Cro	RVC	FNC5	Q	L	271.9	CT
Avemetatarsalia, Aphanosauria	*Teleocrater rhadinus*	Tel	NHMUK PV	R 6795	Q	R	168.5	Ph
Avemetatarsalia, Aphanosauria	*Teleocrater rhadinus*	Tel	NMT	RB 843	Q	R	147.4	SS
Avemetatarsalia, Aphanosauria	*Teleocrater rhadinus*	Tel	NMT	RB 844	Q	R	143.1	SS
Avemetatarsalia, Aphanosauria	*Teleocrater rhadinus*	Tel	NMT	RB 845	Q	R	127.1	SS
Avemetatarsalia, Lagerpetidae	*Kongonaphon kely*	Kon	UA	10618	C	R	38.6	µCT
Avemetatarsalia, Lagerpetidae	*Dromomeron gregorii*	Dro	TMM	31100 464	C	R	91.9	SS
Avemetatarsalia, Lagerpetidae	Dromomeron gregorii*	Dro	TMM	31100 764	C	R	57.3	SS
Avemetatarsalia, Lagerpetidae	Dromomeron gregorii*	Dro	TMM	31100 1308	C	R	81.5	SS
Avemetatarsalia, Dinosauriformes	*Lagosuchus lilloensis*	Lag	PVL	4670	B	R	46.7	CT
Avemetatarsalia, Silesauridae	*Asilisaurus kongwe*	Asi	NMT	RB 159	C	L	140.6	Ph
Avemetatarsalia, Silesauridae	Asilisaurus kongwe*	Asi	NMT	RB 169	C	L	71.4	SS
Avemetatarsalia, Silesauridae	Silesauridae indet.	Sid	TMM	31100 185	C	L	139.8	SS
Avemetatarsalia, Silesauridae	Silesauridae indet.	Sid	TMM	31100 1303	C	L	145.9	SS
Avemetatarsalia, Silesauridae	*Silesaurus opolensis*	Sil	ZPAL	361.23	C	L	192.8	SS
Avemetatarsalia, Ornithischia	*Lesothosaurus diagnosticus*	Les	NHMUK PV	RUB 17	B	R	99.1	SS
Avemetatarsalia, Sauropodomorpha	Mussaurus patagonicus*	Mus	MPM	1813	C	R	114.3	µCT
Avemetatarsalia, Sauropodomorpha	*Mussaurus patagonicus*	Mus	MLP	60 III 20‐22	B	R	814.7	Ph
Avemetatarsalia, Sauropodomorpha	*Plateosaurus* sp.	Pla	GPIT	RE7288	B	R	559.7	CT
Avemetatarsalia, Sauropodomorpha	*Plateosaurus sp*.	Pla	SMNS	13200a+e	B	L	677.9	SS
Avemetatarsalia, Sauropodomorpha	*Plateosaurus sp*.	Pla	SMNS	91300	B	R	614.2	SS
Avemetatarsalia, Sauropodomorpha	*Plateosaurus sp*.	Pla	SMNS	91310	B	L	607.5	SS
Avemetatarsalia, Sauropodomorpha	*Plateosaurus sp*.	Pla	SMNS	91297	B	L	604.8	SS
Avemetatarsalia, Therepoda	*Staurikosaurus pricei*	Sta	MCZ	1699	B	R	220.3	Ph
Avemetatarsalia, Therepoda	*Herrerasaurus ischigualastensis*	Her	MACN	18060	B	L	278.6	SS
Avemetatarsalia, Therepoda	*Herrerasaurus ischigualastensis*	Her	PVL	2566	B	R	435.1	SS
Avemetatarsalia, Therepoda	*Herrerasaurus ischigualastensis*	Her	PVSJ	373	B	L	335.5	SS
Avemetatarsalia, Therepoda	Tawa hallae*	Taw	GR	244	B	R	110.2	SS
Avemetatarsalia, Therepoda	*Tawa hallae*	Taw	GR	1033	B	R	168.5	SS
Avemetatarsalia, Therepoda	*Tawa hallae*	Taw	GR	1054	B	R	202.9	SS
Avemetatarsalia, Neotherepoda	Neotheropoda indet.	Neo	GR	1046	B	R	207.7	SS
Avemetatarsalia, Neotherepoda	*Coelophysis bauri*	Coe	UCMP	129618	B	R	252.7	SS
Avemetatarsalia, Neotherepoda	Coelophysis bauri*	Coe	AMNH	FARB 32843	B	R	124.8	SS
Avemetatarsalia, Neotherepoda	*Dilophosaurus wetherilli*	Dil	UCMP	37302	B	L	586.3	CT
Avemetatarsalia, Avialae	*Archaeopteryx lithographica*	Arc	HMN	1880	B	R	56.9	SS
Avemetatarsalia, Avialae	*Rahonavis ostromi*	Rah	UA	8656	B	L	85.3	µCT

Abbreviations: Abb., used in this study; B, bipedal; C, indeterminate; CT, CT scan; Dig., digitization method; FL, femoral length (mm); L, left; Loc., locomotor mode; Nb., specimen number; Ph, photogrammetry; Q, quadrupedal; R, right; SS, surface scan; µCT, micro‐CT scan. Known juveniles are highlighted with a * after the species name. Patrick O'Connor and colleagues provided access to the *Rahonavis* left femur data, published in conjunction with Forster et al. 2020, with funding from the National Science Foundation. The files were downloaded from www. MorphoSource.org, Duke University; https://doi.org/10.17602/M2/M81891.

Taphonomical alterations impact the original shape of a fossil and consequently alter its biological information (Eifremov, 1940; Webster & Hughes, 1999). Accounting for taphonomy is even more relevant when performing geometric morphometric and statistical analyses, such as here on limb bones (Hedrick et al., 2018; Lefebvre et al., 2020; Pintore et al., 2021 *in press*, Wynd et al., 2021). Thus, only complete femora without clear, visible distortion of the anteroposterior curvature of the diaphysis and of the proximodistal angle between the two epiphyses were considered. Intraspecific parameters such as ontogeny and sexual dimorphism could not be fully accounted for because of the lack of representativeness for each taxon (Mallon, 2017), but known ontogenetic stages are shown in Table 1 (10 out of 69 individuals; Zeigler et al., 2003; Piechowski et al., 2014; Griffin & Nesbitt, 2016; Otero et al., 2019). Originally input locomotor modes indicated in Table 1 are from the adult stage unless otherwise noted (e.g., *Mussaurus*; Otero et al., 2019).

**Institutional Abbreviations**. **AMNH**: American National History Museum, New York, USA; **GPIT**: Geologisch‐Paläontologisches Institut, Tübingen, DE; **GR**: Ghost Ranch Ruth Hall Museum of Palaeontology, Abiquiú, USA; **HMN**: Museum für Naturkunde, Berlin, DE; **MACN**: Museo Argentino de Ciencias Naturales Bernardino Rivadavia, Buenos Aires, AR; **MCZ**: Museum of Comparative Zoology, Harvard University, Cambridge, USA; **MLP**: Museo de La Plata, La Plata, AR; **MPM**: Museo Regional Provincial “Padre M. J. Molina,” Santa Cruz, AR; **NHMUK PV**: Natural History Museum, London, UK; **NMMNH**: New Mexico Museum of Natural History and Science, Albuquerque, USA; **NMT**: National Museum of Tanzania, Dar es Salaam, TZ; **PEFO**: Petrified Forest National Park, Arizona, USA; **PVL**: Paleontología de Vertebrados, Instituto Miguel Lillo, Tucumán, AR; **PVSJ**: División de Paleontología de Vertebrados del Museo de Ciencias Naturales y Universidad Nacional de San Juan, San Juan, AR; **RVC**: Royal Veterinary College, Hatfield, UK; **SAM PK**: Iziko South African Museum, Cape Town, ZA; **SMNS**: Staatliches Museum für Naturkunde, Stuttgart, DE; **TMM**: Jackson School of Geosciences Vertebrate Paleontology Laboratory, University of Texas, Austin, USA; **TTUP**: Texas Tech University Museum, Lubbock, USA; **UCMP**: University of California Museum of Paleontology, Berkeley, USA; **UA**: Université d’Antananarivo, Antananarivo, MG; **ZPAL**: Institute of Paleobiology of the Polish Academy of Sciences, Warsaw, PL.

### 2.2 3D digitization

Fossils were digitized using different approaches (Table 1). Most specimens were digitized with photogrammetry using a Nikon D550 camera (Nikon Inc.) with lenses of 18‐55mm and 50mm depending on their size (20 out of 72 specimens). Specimens were placed on a turntable in a light tent in order to avoid artefacts caused by lighting and positions. 3D reconstructions were performed using Agisoft Metashape Professional v. 1.6.1 10009 (Agisoft LLC) to create dense clouds and align specimens and Meshlab v. 2020.06 (Cignoni et al., 2008) to create and scale meshes. Different surface scanners (32 out of 72 specimens) were also used: NextEngine (NextEngine Inc.) with ScanStudio Pro v. 2.0.2 (NextEngine Inc.) for the reconstruction; or Artec EVA and Space Spider (Artec 3D, Luxembourg) with Artec Studio Professional v. 12.1.1.12 (Artec 3D, 2018). Some specimens were scanned using CT and micro‐CT scanners (20 out of 72 specimens; *Plateosaurus*: Mallison, 2010; also Table 1, S1). Mimics v. 23 software (Materialise NV, Leuven, Belgium) was used to segment CT and micro‐CT scans in order to create meshes. Blender v. 2.8 (The Blender Foundation) was used to mirror left femora and to re‐assemble fragmentary bones based on contacts between matching surfaces. Past analyses showed that surface scans, photogrammetry, and CT scans produced 3D reconstructions with similar quality (Falkingham, 2012; Fau et al., 2016), which is especially true for large specimens at the resolution reached here. Furthermore, Soodmand et al. (2018) showed that there was no significant difference between 3D models of a femur digitized with both CT and surface scanner; and Dìez Dìaz et al. (2021) showed that, despite the superior visual quality of photogrammetry, the difference in the geometry of 3D meshes generated from photogrammetry and an Artec EVA scanner was even lower than reported in Fau et al. (2016) (e.g., <0.01 mm against 0.6 mm). Finally, Waltenberger et al. (2021) showed that osteological 3D models obtained from surface scans, photogrammetry, and CT scans could be combined in a single analysis when using 3D GMM.

### 2.3 Geometric morphometrics

Femoral shape variation was investigated with 3D GMM. This approach is well suited to biology and palaeontology as it measures the variation between different biological shapes using spatial markers with correspondence between homologous anatomical locations on every specimen (Zelditch et al., 2012). Anatomical landmarks and sliding semilandmarks on curves and surfaces were digitized following the protocol of Gunz et al. (2005), Gunz and Mitteroecker (2013), and Botton‐Divet et al. (2016). Anatomical landmarks on limb bones are usually concentrated only on ends and were reported to not effectively capture the shape variation along the diaphysis (Botton‐Divet et al., 2016). Sliding semilandmarks are suited to circumvent the lack of anatomical landmarks on the diaphysis because they are placed in spatially homologous positions. Moreover, high‐density GMM is more effective at accurately capturing the shape variation between biological objects than anatomical landmarks alone (Botton‐Divet et al., 2015; Goswami et al., 2019; Gunz & Mitteroecker, 2013; Gunz et al., 2009; Zelditch et al., 2012).

**FIGURE 2:**
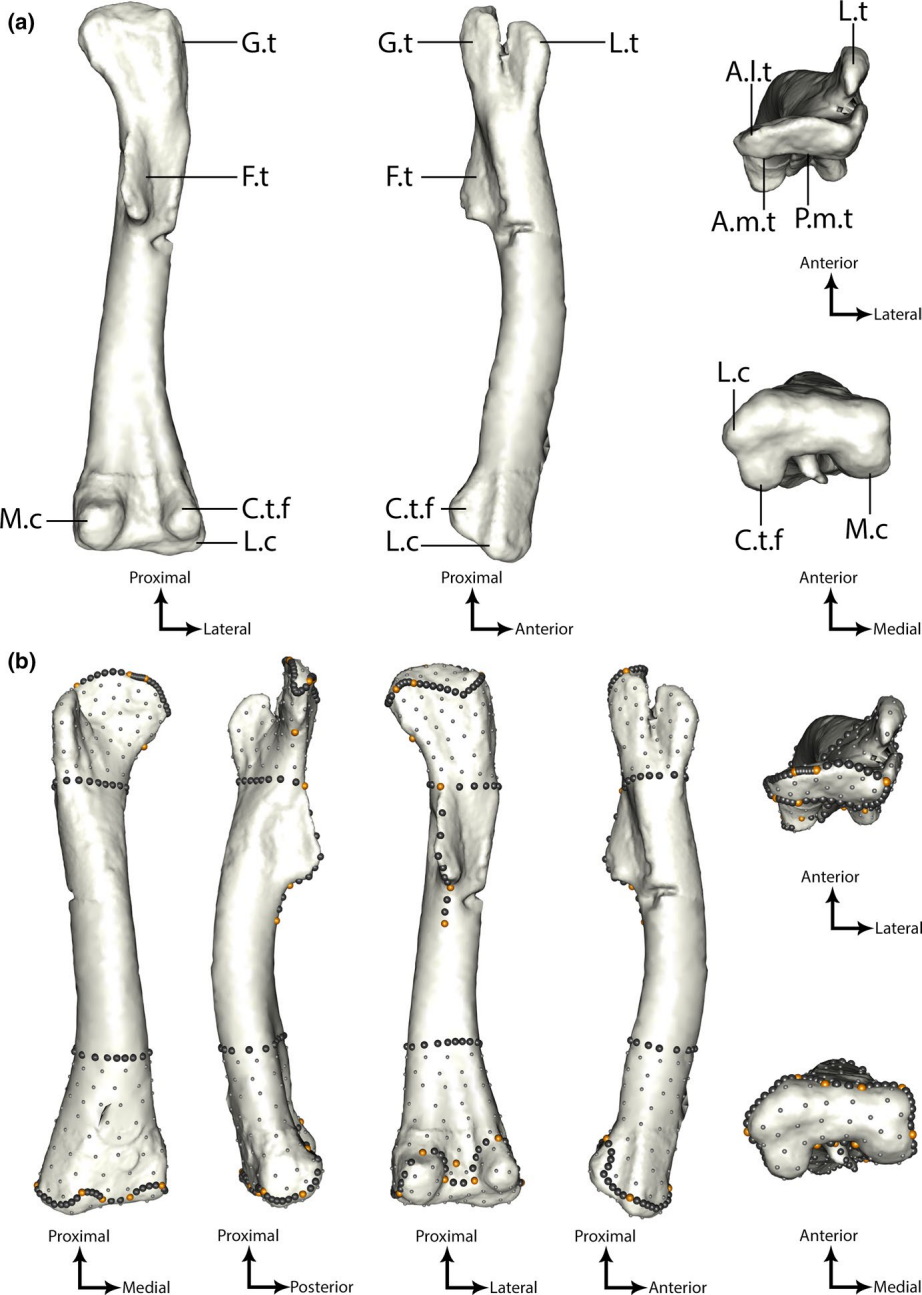
Template right femur of *Lesothosaurus* (NHMUK PV RUB17): (a) without landmarks; (b) with anatomical landmarks (orange) and sliding semilandmarks on curves (black) and surfaces (grey). A.l.t, anterolateral tuber; A.m.t, anteromedial tuber; C.t.f, crista tibiofibularis; F.t, fourth trochanter; L.t, lesser trochanter; M.c, medial condyle; P.m.t, posteromedial tuber

Four hundred and twenty‐five landmarks including 20 anatomical landmarks, 176 sliding semilandmarks on curves, and 229 on surfaces were digitized on each specimen using IDAV Landmark software (Wiley et al., 2005 v. 3.0.0.6). Anatomical landmarks and sliding semilandmarks on curves were manually digitized on every specimen by relying on concavities rather than convexities along anatomical features when possible in order to minimize the impact of taphonomy along eroded features (Table S2). Additionally, sliding semilandmarks on surfaces were manually digitized on one chosen specimen referred as the “template” (Figure S1). We chose the femur of *Lesothosaurus* as the template because it has the most prominent and consistent features of the sample, ensuring that sliding semilandmarks would be correctly projected on other femora (Figure 2b). Next, sliding semilandmarks on surfaces were automatically projected onto all other specimens by performing a series of spline relaxations that minimized the bending energy of a Thin Plate Spline (TPS), using the R package Morpho v. 2.8 (Schlager, 2017). During this first step, a spline relaxation was performed between sliding semilandmarks on template curves and every other specimen. The interpolation of semilandmarks on curves was then used to project semilandmarks from the template surfaces onto every other specimen surfaces using the function “placePatch” of the Morpho package. The second step was to perform five iterations of another spline relaxation between the complete landmark configuration of the template and the ones from each specimen, using the function “relaxLM” of Morpho. The last step was to compute a Procrustes consensus of every configuration using a partial Procrustes fitting and use that as a reference to perform a final spline relaxation between every specimen in two iterations, using the function “slideLM” of Morpho. These three steps ensured that every semilandmark position was geometrically homologous between every specimen and could be interpreted consistently with anatomical landmark displacements (Gunz et al., 2005).

In addition, two curves were digitized using semilandmarks around the circumference at the most proximal and distal parts of the shaft in order to restrict surface semilandmarks only to the proximal and distal ends of the bones. This was done to limit the impact of taphonomic alteration caused by the distortion/crushing of the diaphysis on the calculation of the shape variation. We removed specimens with too much damage to the shaft area (e.g., unusual orientation of the shaft based on well‐preserved specimens from the same/closely related taxon). These two curves were digitized using the most proximal landmark on the fourth trochanter for the proximal part and a geometrical criterion—the point of abrupt change in shaft circumference above the distal end—on the distal part (Figure 2b). These two curves were then removed after the sliding landmark procedure and before computing the shape analysis, and thus not included in the analysis, to avoid biasing measures of shape difference. However, we found that delimitation curves had little to no impact on the main results when we integrated them in a parallel analysis (Figure S2).

A Generalized Procrustes Analysis (GPA) was performed on the resulting landmark configurations of every specimen in order to homogenize their positions in the Cartesian coordinate system by superimposing them (Gower, 1975; Rohlf & Slice, 1990), using the function “gpagen” of the geomorph v. 3.3.1 R package (Adams & Otárola‐Castillo, 2013). This step also enabled us to isolate the shape component from the size component (Zelditch et al., 2012). It was hence possible to study shape variation for every specimen by focusing on Procrustes residuals once the GPA was performed.

A Principal Component Analysis (PCA) was then computed in order to reduce dimensionalities of the variation (Gunz & Mitteroecker, 2013) and notably to identify if one axis would polarize the morphological variation linked to different locomotor modes based on “known” bipedal and quadrupedal archosauriforms (Table 1). Repeatability testing was performed by digitizing anatomical landmarks iteratively (*n* = 10) on the three *Postosuchus* femora, which was one of the taxa with the least intraspecific variability within the sample when more than one specimen was available. A PCA was then computed, which showed that all landmark configurations of the same specimen were grouped together and isolated from those of the other specimens along the two first PC axes (Figure S3), meaning that the biological variability was greater than the operator effect (e.g., the ability to reproduce the same landmark configuration multiple times on the same specimen).

Convex hulls were used in order to highlight the distribution of locomotor modes in the morphospace using the function “shapeHulls” from the geomorph v. 3.3.1 R package (Adams & Otárola‐Castillo, 2013). Isolating the shape variation linked to differences in locomotor mode also enabled us to compute 3D visualizations that highlighted which features varied the most along this axis. This was done by computing a mean shape between all the specimens of the sample. The mean shape was created by performing a spline relaxation between the template landmark configuration and a mean landmark configuration that was obtained after the GPA was performed. The resulting TPS deformation was used to deform the template mesh into a mean shape of all the specimens. The mean shape was then interpolated again with landmark configurations associated to the positive and negative extremes of the selected axis to create minimal and maximal theoretical shapes. This procedure also enabled us to quantify how much femoral features scored in cladistic analyses (e.g., Nesbitt, 2011) varied relative to another along each PCA axis. Vectors of displacement between every landmark of the two theoretical shapes were computed using the function “segments3d” of the rgl v. 0.100.54 R package (Adler & Murdoch, 2020). A gradient of color was applied to these segments according to the distance between each landmark in order to highlight which parts varied the most (Botton‐Divet, 2017). This gradient was computed by using the “blue2red” function of the ColorRamps R package (Keitt, 2008). OnScreenProtractor v. 0.5 (GNU GPLv3) was used to measure the angle between features relying on two anatomical landmarks in the medio‐lateral axis on each bone end (Figure S4). We deliberately chose to constrain our linear morphometrics analysis to the pre‐existing landmarks we used in the geometric morphometrics analysis in order to ensure that our results remained comparable. Resulting measures were shown using boxplots computed in ggplot2 v. 3.3.2 (Wickham, 2016).

The allometric effect—or the size‐related morphological variation across both evolution (i.e., evolutionary allometry) and ontogeny (ontogenetic allometry; Klingenberg, 2016)—was computed after the GPA and the PCA. We first conducted a Pearson's correlation test between the log‐transformed centroid size of each specimen and their distribution along the chosen PC axis within the morphospace using the R function “cor.test.” A significant result would indicate that shape variation along that axis had an allometric component. While Mitteroecker and Gunz (2009) stated that “the regression of shape on the logarithm of centroid size is the most optimal measure for allometry (p. 243)”, Campione and Evans (2012, 2020) demonstrated that the minimum diaphyseal circumference (MDC) of the femur was a reliable predictor for body size in non‐avian dinosaurs and quadrupedal terrestrial tetrapods. Therefore, we performed a correlation analysis between log‐transformed centroid sizes and log‐transformed MDC in order to test if it was reliable to use the femoral centroid size as a proxy for body size (e.g., body mass). We measured the MDC across all specimens from our sample (Table S3) using the “cross section” and “extract contours” tools from the software CloudCompare 2.12 alpha (http://www.cloudcompare.org). We then computed a correlation test and a regression plot using the R function “lm.”

A phylogeny was constructed following the dataset of Nesbitt (2011) and recent iterations (Butler et al., 2014; Ezcurra et al., 2020) using Mesquite software v. 3.61 (Maddison & Maddison, 2019) with all branch lengths set to 1. The phylogenetic position of *Parringtonia* within Archosauria remains poorly understood (Foffa et al., 2020; Nesbitt & Butler, 2013). However, we followed the finding of Nesbitt et al. (2018) that *Parringtonia* was an early diverging suchian based on its braincase anatomy. The phylogenetic position of *Nundasuchus* is also uncertain (Butler et al., 2017; Ezcurra, 2016; Nesbit, 2011; Nesbitt et al., 2014; Roberto‐Da‐Silva et al., 2018). Thus, we chose to follow the phylogeny of Nesbitt et al. (2014) and Butler et al. (2017), but it could be closer to the base of Pseudosuchia (see Ezcurra et al., 2020). Using this constructed phylogeny (Figure 1), a phylomorphospace was computed using geomorph with the function “plot.gm.prcomp” with the argument “phylo” set to “TRUE.” The K_mult_ statistic was used in order to quantify phylogenetic implication in the shape variation using the function “physignal” of the same R package. The K_mult_ statistic is a multivariate extension of the K statistics from Blomberg et al. (2003), which is adapted to a multivariate dataset (Adams, 2014a). Its calculation relies on comparisons between the “actual” phylogeny and expectations under a Brownian motion model of evolution based on the distribution of specimens across the morphospace. When significant, the value of K_mult_ >1 suggests that the distribution of femoral shape across the morphospace varies between clades and within a clade when K_mult_ <1 (Adams, 2014a). We also performed a Phylogenetic Generalized Least Squares (PGLS) regression to test the influence of size and locomotor mode on femoral morphology in a phylogenetic context under a Brownian motion model of evolution using the function “procD.pgls” of the geomorph package (Adams, 2014b).

A k‐nearest neighbors (k‐NN) analysis was performed along the second PC axis, subsequently identified as linked to locomotor mode using Procrustes distances in order to determine locomotor modes of indeterminate specimens based on femoral shape and the “known” attribution of their closest neighbors. The function “knn” of the class R package (Venables & Ripley, 2002) was performed iteratively for each indeterminate specimen with the number of closest neighbors set to five.

## 3 RESULTS

### 3.1 Principal component analysis

**FIGURE 3:**
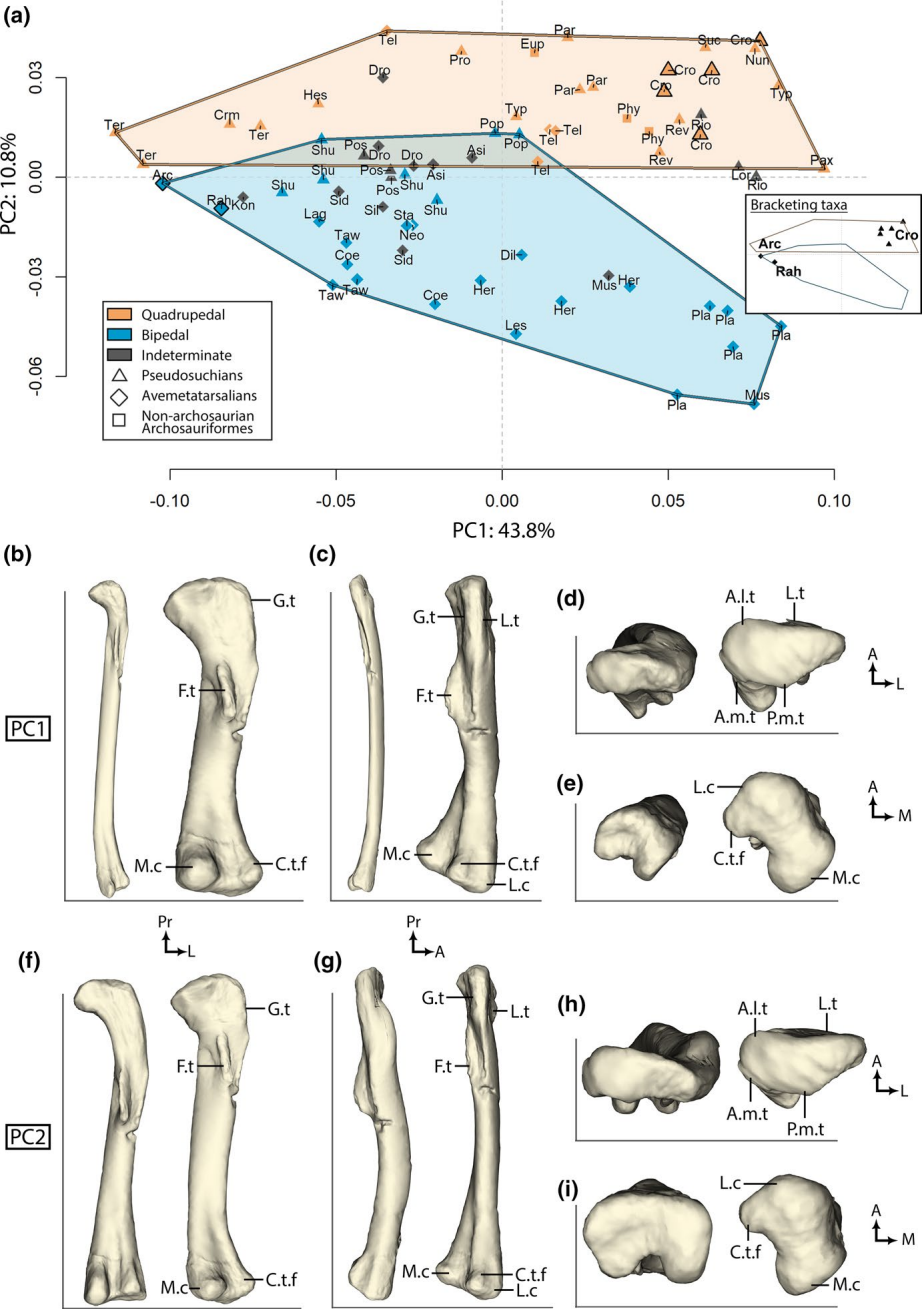
(a) PCA with cluster of locomotor mode. Bracketing taxa from after the Early Jurassic (*Crocodylus* and avialan dinosaurs) are outlined in bold and in the small PCA panel. Taxonomic abbreviations: see Table 1. Minimal (left) and maximal (right) theoretical (interpolated) shapes for PC1 (b, c, d, e) and PC2 (f, g, h, i) in (b, f) posterior, (c, g) lateral, (d, h) proximal, and (e, i) distal views. A, anterior; A.l.t, anterolateral tuber; A.m.t, anteromedial tuber; C.t.f, crista tibiofibularis; F.t, fourth trochanter; L, lateral; L.t, lesser trochanter; M, medial; M.c, medial condyle; P.m.t, posteromedial tuber; Pr, proximal

The first two axes (PC1, PC2) accounted for more than 50% of the global shape variation (43.8% and 10.8%, respectively; Figure 3a). PC1 represented femoral robusticity whereas PC2 broadly represented different femoral angulations. Bipedal and quadrupedal archosauriforms were not sorted along the first axis (PC1) because both groups occupied the whole morphospace (Figure 3a). The femur of *Terrestrisuchus* (a lightly built, presumably quadrupedal Triassic crocodylomorph) was the taxon with the most negative value on PC1, and *Paratypothorax* (a heavily built quadrupedal aetosaur) had the most positive value (Figure 3a). PC1 was linked to the increase of epiphyseal width relative to femoral length (i.e., femoral robusticity; Figure 3b‐e), as demonstrated by the theoretical shapes at its extremes. The theoretical shape at the negative extreme of PC1 had relatively smaller ends (epiphyses)—especially along the mediolateral and proximodistal axes—and a narrower shaft than the theoretical shape at the positive extreme (Figure 3b‐e). The fourth trochanter (Figure 2) was flatter and closer to the proximal end in the theoretical shape at the negative extreme and more rounded and closer to the middle part of the shaft in the theoretical shape at the positive extreme (Figure 3b,c). The crista tibiofibularis and the lateral and medial condyles of the distal end of the femur were more prominent on the minimal theoretical shape than on the maximal one (Figure 3e).

Quadrupedal archosaurs formed a cluster together on the positive side of PC2 and bipedal archosaurs on the negative one, with a small overlap involving the two femora of the same specimen of the bipedal pseudosuchian *Poposaurus* and only one specimen of *Shuvosaurus* (out of five), as well as one specimen of the presumably quadrupedal pseudosuchians *Terrestrisuchus* (out of three), *Teleocrater* (out of four), *Revueltosaurus* (out of two), and *Paratypothorax* (Figure 3a; “Pop, Shu, Ter, Tel, Rev, Par”). One specimen of *Teleocrater* was the specimen with the most positive value on the PC2 axis and adult *Mussaurus* (heavily built, presumably bipedal sauropodomorph dinosaur) was the specimen with the most negative value (Figure 3a; “Tel, Mus”). The bipedal cluster had a wider extent than the quadrupedal one along PC2, notably with large sauropodomorphs extending the distribution the furthest away from quadrupedal taxa (Figure 3a). Similarly, the separation between bipedal and quadrupedal clusters increased toward the positive side of PC1, whereas specimens on the negative side of PC1 showed no clear separation, and did not extend so far negatively on PC2 (Figure 3a). Minimal and maximal theoretical shapes along PC2 showed that bones were anteroposteriorly more curved on the negative side (Figure 3g). The proximodistal twist or offset between the two epiphyses was greater in the maximal shape than in the minimal one (Figure 3h,i). Furthermore, the fourth trochanter was more rounded, and the area around the lesser trochanter was more prominent on the minimal theoretical shape than on the maximal one (Figure 3f,g).

The k‐NN performed on the Procrustes distances along PC2 showed that 93.1% of specimens with “known” locomotor mode were correctly predicted (Table 2). The remaining 6.9% that were wrongly predicted were *Paratypothorax* and the two *Poposaurus* and two *Shuvosaurus* specimens (Table 2). Specimens with indeterminate locomotor modes represented 22% of the whole sample. Predicted locomotor modes showed variation among the same species for *Postosuchus*, *Riojasuchus*, and *Dromomeron*, whereas predictions were consistent across all specimens of the same species for *Poposaurus*, *Asilisaurus*, and *Mussaurus* and across the same clade for Silesauridae. Moreover, predictions were consistent between left/right femora from the same individual of *Postosuchus* and *Poposaurus* (Table 2).

**TABLE 2:** Estimated locomotor habits based on the k‐NN results performed along PC2

Name	Input locomotor mode	k‐NN PC2
*Archaeopteryx* HMN 1880	B	B
***Asilisaurus* NMT RB159**	**I**	**Q**
***Asilisaurus* NMT RB169**	**I**	**Q**
*Coelophysis* AMNH FARB 32843	B	B
*Coelophysis* UCMP 129618	B	B
*Crocodylus* DDNC01	Q	Q
*Crocodylus* DDNC02	Q	Q
*Crocodylus* DDNC03	Q	Q
*Crocodylus* DDNC04	Q	Q
*Crocodylus* FNC5	Q	Q
*Dilophosaurus* UCMP 37302	B	B
***Dromomeron* TMM 31100‐464**	**I**	**B**
***Dromomeron* TMM 31100‐764**	**I**	**Q**
***Dromomeron* TMM 31100‐1308**	**I**	**Q**
*Euparkeria* SAM‐PK‐5867	Q	Q
*Herrerasaurus* MACN 18060	B	B
*Herrerasaurus* PVL 2566	B	B
*Herrerasaurus* PVSJ 373	B	B
*Hesperosuchus* AMNH FR 6758	Q	Q
***Kongonaphon* UA 10618**	**I**	**B**
*Lesothosaurus* NHMUK RUB17	B	B
*Lagosuchus* PVL 4670	B	B
*Mussaurus* MLP60‐III‐20‐22	B	B
***Mussaurus* MPM 1813**	**I**	**B**
Neotheropoda GR1046	B	B
*Nundasuchus* NMT RB48	Q	Q
***Paratypothorax* TTU‐P12547**	**Q**	**B**
*Parringtonia* NMT RB188	Q	Q
*Parringtonia* NMT RB426 (L)	Q	Q
*Parringtonia* NMT RB426 (R)	Q	Q
Phytosauridae PEFO 23347	Q	Q
Phytosauridae PEFO 31219	Q	Q
*Plateosaurus* GPIT RE7288	B	B
*Plateosaurus* SMNS 13200a+e	B	B
*Plateosaurus* SMNS 91297	B	B
*Plateosaurus* SMNS 91300	B	B
*Plateosaurus* SMNS 91310	B	B
***Poposaurus* YPM 57100 (L)**	**B**	**Q**
***Poposaurus* YPM 57100 (R)**	**B**	**Q**
***Postosuchus* TTU‐P9000**	**I**	**B**
***Postosuchus* TTU‐P9002 (L)**	**I**	**Q**
***Postosuchus* TTU‐P9002 (R)**	**I**	**Q**
*Protosuchus* AMNH FR 3024	Q	Q
*Rahonavis* UA8656	B	B
**Loricata NMMNH P‐36144**	**I**	**Q**
*Revueltosaurus* PEFO 34269	Q	Q
*Revueltosaurus* PEFO 34561	Q	Q
***Riojasuchus* PVL 3827**	**I**	**B**
***Riojasuchus* PVL 3828**	**I**	**Q**
*Shuvosaurus* NMMNHP‐4695	B	B
***Shuvosaurus* TTU‐P18307**	**B**	**Q**
*Shuvosaurus* TTU‐P18321	B	B
*Shuvosaurus* TTU‐P18336	B	B
***Shuvosaurus* TTU‐P9001**	**B**	**Q**
**Silesaurid TMM 31100–1303**	**I**	**B**
**Silesaurid TMM31100‐185**	**I**	**B**
***Silesaurus* ZPAL361.23**	**I**	**B**
Sphenosuchian TTU‐P11443	Q	Q
*Staurikosaurus* MCZ 1699	B	B
Suchian NMT RB187	Q	Q
*Tawa* GR 1033	B	B
*Tawa* GR 1054	B	B
*Tawa* GR 244	B	B
*Teleocrater* NHMUK PV R6795	Q	Q
*Teleocrater* NMT RB843	Q	Q
*Teleocrater* NMT RB844	Q	Q
*Teleocrater* NMT RB845	Q	Q
*Terrestrisuchus* 721.3	Q	Q
*Terrestrisuchus* R10002	Q	Q
*Terrestrisuchus* Composite	Q	Q
*Typothorax* NMMNH‐P11775	Q	Q
*Typothorax* NMMNH‐P11778	Q	Q

Note

Taxa with different attributions than the originally input one are highlighted in bold. Abbreviations: B, bipedal; I, indeterminate; Q, quadrupedal; L, Left; R, Right.

### 3.2 Morphological variation

**FIGURE 4:**
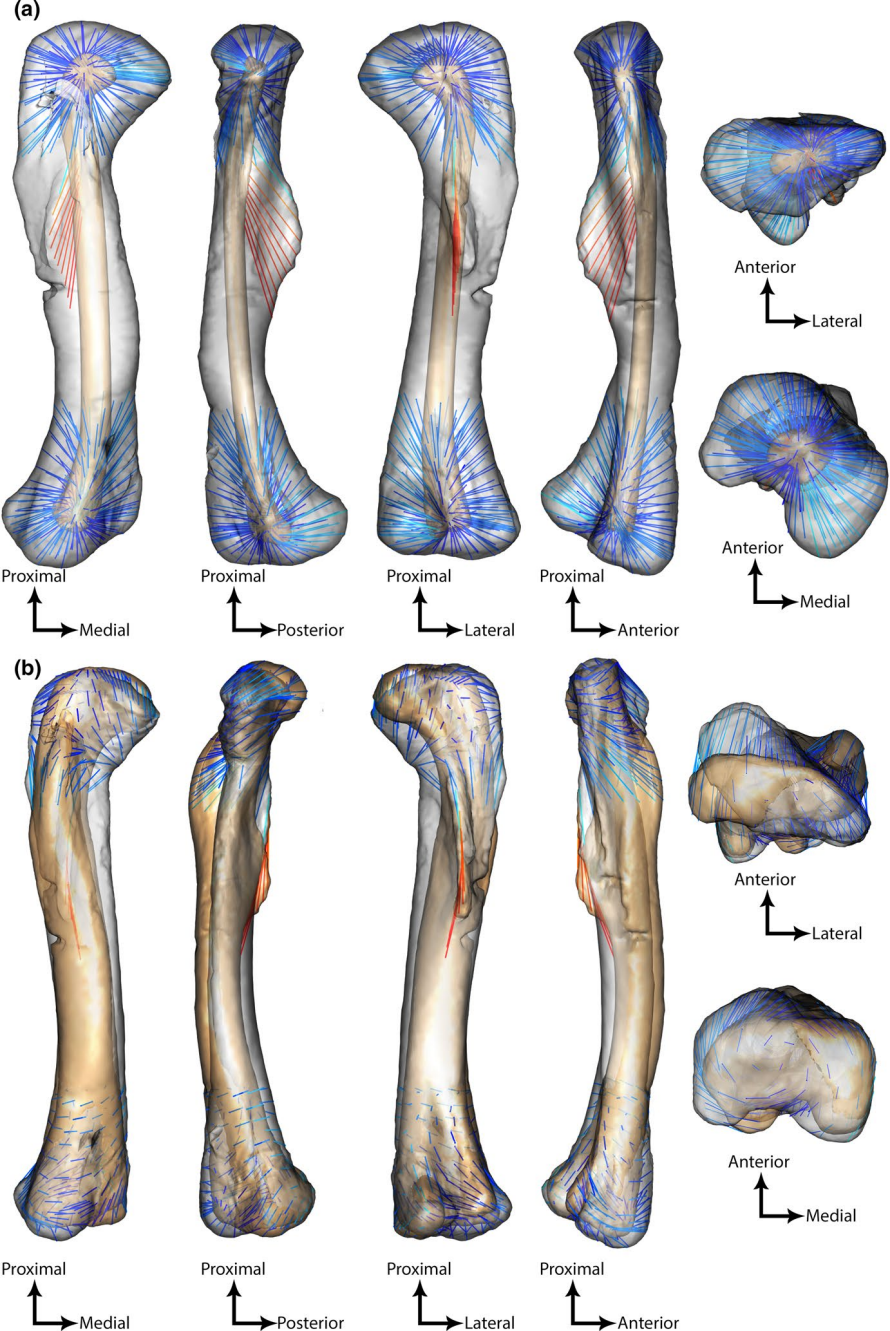
Morphological variation between minimal (colored) and maximal (grey) theoretical (interpolated) shapes along a) PC1 and b) PC2 in anterior, medial, lateral, posterior, proximal, and distal views. Vectors showing landmark displacements are shown with colors ranging from cold (low distance) to hot (high distances)

The shape variation between extremes of each axis was quantified using colored vectors between corresponding landmarks (Figure 4a,b). This visualization allowed us to highlight that the fourth trochanter was the feature varying the most along PC1 and PC2 (Figure 4a,b).

Along PC1, vectors between landmarks were longer on the distal part of the fourth trochanter than on the proximal one (Figure 4a). This showed that the fourth trochanter was longitudinally larger toward its distal end on robust femora than it was on gracile femora (Figure 4a). We interpret this to indicate that the fourth trochanter was located more proximally along the shaft of gracile femora (i.e., was distally smaller) than on more robust ones (Figures 3c, 4a). The fourth trochanter had a more rounded shape on robust femora and a flatter ridge on the more gracile ones (Figure 4a). Additionally, the proximal and distal ends of robust femora were mostly wider along the mediolateral axis than in gracile femora, with a thicker medial condyle and crista tibiofibularis (Figure 4a).

**FIGURE 5:**
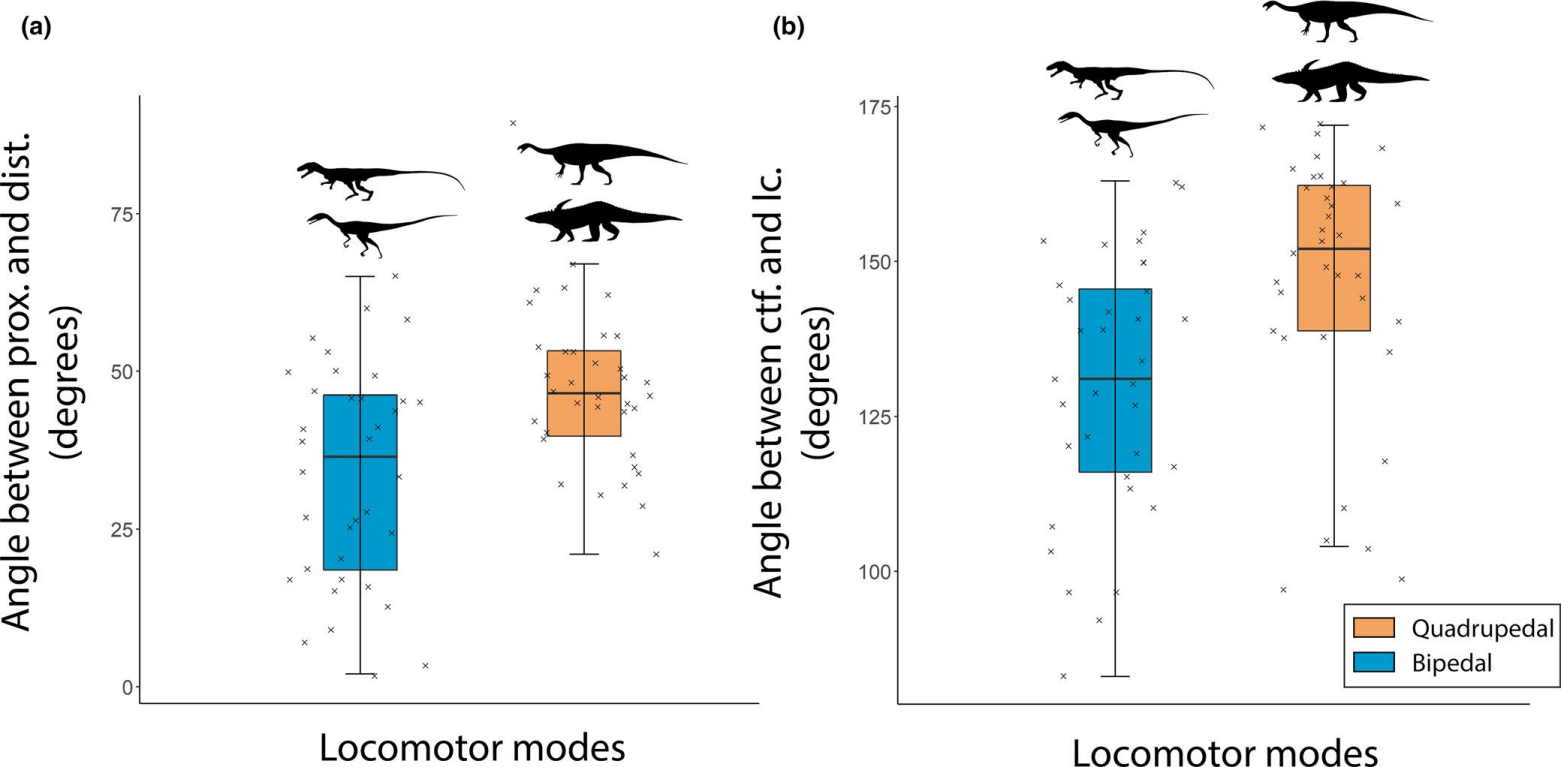
Boxplots for bipedal (blue) and quadrupedal (orange) archosauriforms showing angles between the (a) longest proximal (prox.) axis in relative to the distal (dist.) one for the femoral head vs. epiphyses (smaller angle = more medially offset femoral head), (b) crista tibiofibularis (ctf.), and lateral condyle (lc.) (smaller angle = more laterally offset ctf.). Silhouettes: Bipedal = top, *Poposaurus* (modified after Schachner et al., 2019); bottom, *Tawa* (modified after Nesbitt et al., 2009); Quadrupedal = top, *Plateosaurus* (modified after Hartman S. 2013; thought to be bipedal but shown here simply as a large, early sauropodomorph as some of these may have been quadrupedal); bottom, *Desmatosuchus* (modified after Parker & Martz, 2011)

Along PC2, the fourth trochanter was sharp and symmetrical for quadrupeds and rounded and asymmetrical with a steep slope in the distal part for bipeds (Figure 4b). Landmarks on the fourth trochanter were displaced mostly along a proximodistal axis, meaning that the prominence of this feature did not vary much along the anteroposterior axis (Figure 4b). The femoral head's orientation with respect to the distal end (i.e., medial “twisting” or offset of the head vs. epiphyses) was also one of the greatest morphological variations, with the median angle close to 45° on bipeds’ femora and greater (less medially oriented) on quadrupeds’ femora (Figures 4b, 5a, Table S3). In distal view, the angle between the lateral condyles and crista tibiofibularis was greater on femora from quadrupeds than those from bipeds (Figures 4b, 5b, Table S3). The mean measured angles for bipeds and quadrupeds (from k‐NN PC2 results in Table 2) were significantly different for both femoral head rotation and the angle between the lateral condyle and the crista tibiofibularis (the sample was normally distributed because there were more than 30 individuals, variables were independents and equal [two‐variances F‐test: *p*‐value >0.05], and there was a significant difference in means between the two samples [*T*‐test: *p* < 0.01]). Finally, the lesser trochanter was more prominent along the anteroposterior axis in the femora of bipeds than femora of quadrupeds (Figure 4b). More specifically, the distal part of the lesser trochanter was the region with the greatest landmark displacements (Figure 4b).

### 3.3 Phylogenetic signal

**FIGURE 6:**
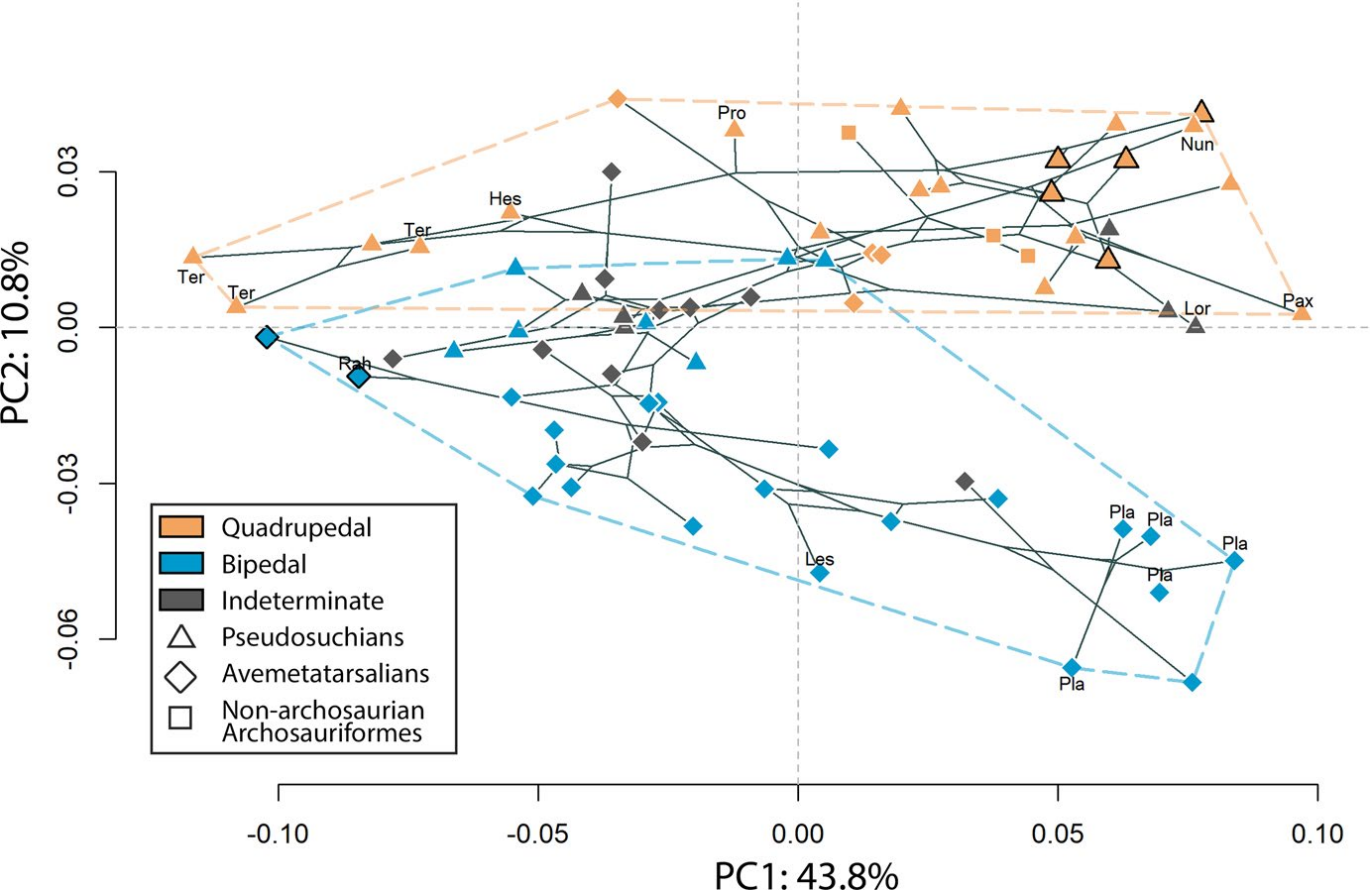
Phylomorphospace with branches mapped onto the PCA (see Figure 3a). Convex hulls follow the same color code. Black outline: Bracketing taxa from after the Early Jurassic (*Crocodylus* and avialan dinosaurs) are outlined in bold (see panel in Figure 3). Labels are the same as in Figure 3 but fewer nodes are labeled for clarity

Results from the multivariate K statistic were all significantly correlated with phylogeny. However, K_mult_ was below one when calculated for the global morphological variation (K_mult_: 0.46, *p* < 0.01) and for the variation along PC1 (K: 0.57, *p* < 0.01), meaning that the morphological variation was structured within clades (Figure 6). Conversely, K was clearly above one when calculated along PC2 (K: 1.85, *p* < 0.01), meaning that the morphological variation isolated along PC2 varied between clades (Figure 6).

The phylomorphospace illustrated the K statistics, with the distribution along PC2 clearly varying between clades whereas the distribution along PC1 varied within clades (Figure 6). Both avemetatarsalians (*Plateosaurus* and *Mussaurus*) and pseudosuchians (aetosaurs, ornithosuchians, *Crocodylus*, *Nundasuchus*, early diverging suchian NMT RB187 and loricatan NMMNH P‐36144), along with Phytosauridae, displayed comparable levels of femoral robusticity (Figure 3a “Pla, Mus, Typ, Par, Rio, Cro, Nun, Suc, Lor, Phy,” 6). Apart from the two avialan theropods, mostly non‐crocodylian crocodylomorphs (*Terrestrisuchus*, *Hesperosuchus*, crocodylomorph TTU‐P11443) were represented on the most negative side of PC1, along with the small lagerpetid *Kongonaphon* (Figure 3a; “Ter, Hes, Crm, Kon,” 6). Smaller bipedal theropod dinosaurs displayed similar slightly negative values along PC1 compared with *Dromomeron*, *Lagosuchus*, Silesauridae, *Euparkeria*, *Shuvosaurus*, *Poposaurus*, *Postosuchus*, and *Protosuchus* (Figure 3a; “Dro, Lag, Eup, Shu, Pop, Pos, Pro,” 6). The larger theropods *Dilophosaurus* and *Herrerasaurus* had slightly positive values along PC1 along with *Teleocrater*, *Parringtonia*, and the small ornithischian *Lesothosaurus* (Figure 3a; “Dil, Her, Tel, Par, Les,” 6). Thus, the variation of femoral robusticity varied within clades because some pseudosuchians and some avemetatarsalians displayed similar levels of femoral robusticity (Figures 3a, 6).

The major variation between clades was along PC2, with pseudosuchians having mostly positive values whereas avemetatarsalians had mostly negative values (Figure 6). *Euparkeria* and phytosaurs also displayed positive values (Figure 3a; “Eup, Phy,” 6). This distinction did not apply to the early avemetatarsalians *Teleocrater*, *Dromomeron*, and *Asilisaurus* which had positive values, as well as the pseudosuchian *Shuvosaurus* for which half of the specimens had negative values (Figure 3a; “Tel, Dro, Asi, Shu,” 6).

### 3.4 Evolutionary allometry

**FIGURE 7:**
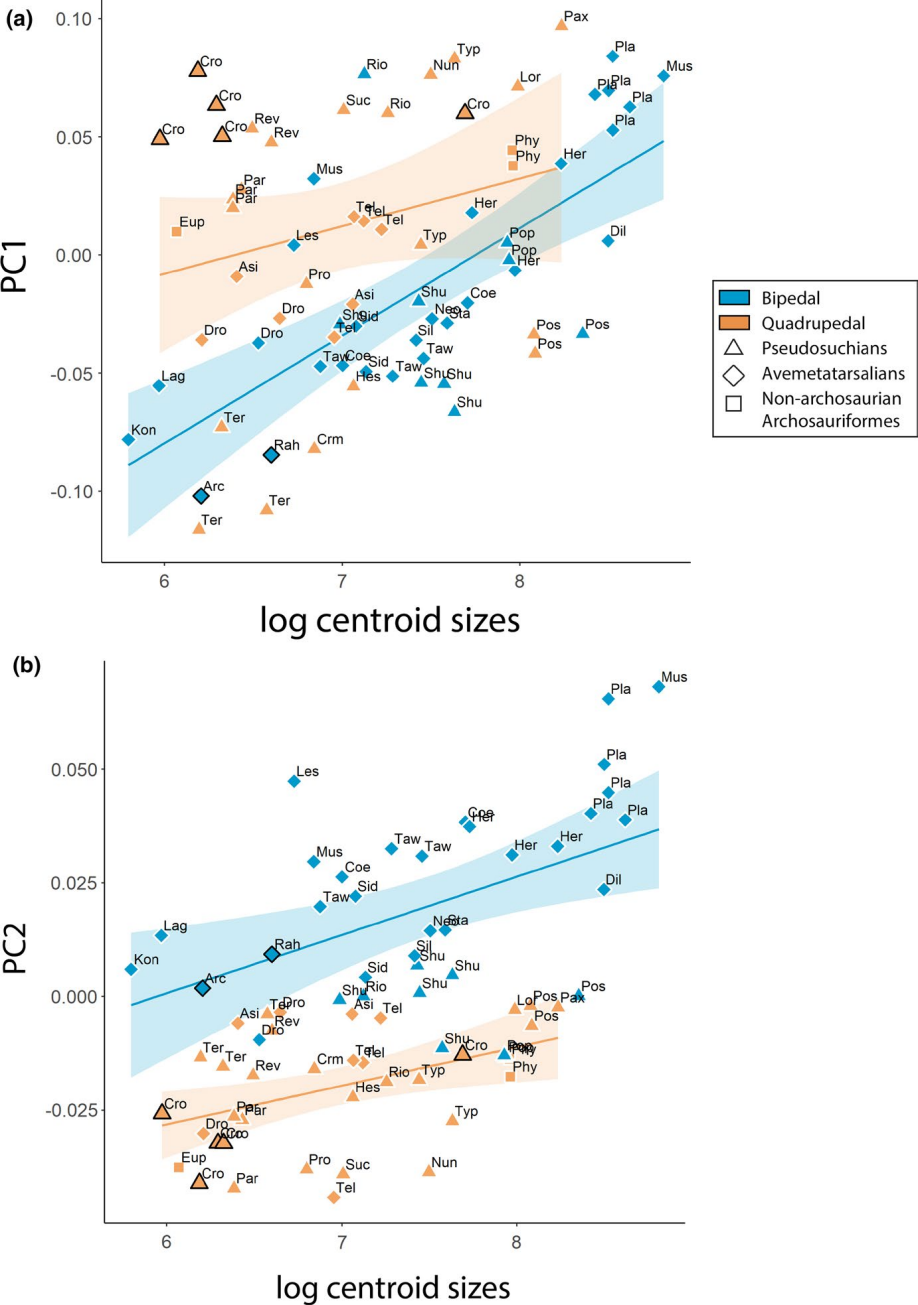
Regression between log‐transformed centroid sizes and (a) PC1, (b) PC2. Bracketing taxa from after the Early Jurassic (*Crocodylus* and avialan dinosaurs) are outlined in bold

First, we found a strong association between log‐transformed minimal diaphyseal circumference (MDC) and log‐transformed centroid sizes (*r²*: 0.9, *p* < 0.01; Figure S5), indicating that centroid sizes, at least from femoral morphology, can be reliably used as an indicator of body size. Secondly, we found a significant but small impact of size (log‐transformed centroid sizes) on femoral morphology when we performed the PGLS accounting for every PC axis (*r²*: 0.1, *p* < 0.01) and a significant but even smaller impact of locomotor habit (including estimated locomotor modes; *r²*: 0.03, *p* < 0.05), with no significant interaction between size and locomotor modes (*r²*: 0.02, *p* > 0.05). The PGLS enabled a general overview of the interaction between shape, size, and locomotor variables at a multidimensional level when factoring out phylogeny, but studying the femoral shape at the unidimensional level along the selected axis as well completed our understanding of these interactions. We focused on the strongest association between femoral robusticity, which represented 43.8% of the global variation at least along PC1, and centroid size while accounting for locomotor habit. Log‐transformed centroid sizes were positively correlated with the distribution along PC1, meaning that centroid size increased toward the positive side of PC1 (*r²*: 0.13, *p* < 0.01, Figure 3a). Conversely, log‐transformed centroid sizes were negatively correlated with the distribution along PC2, meaning that centroid sizes decreased toward the positive side of PC2 (*r²*: 0.29, *p* < 0.01, Figure 3a). Thirdly, we found that this apparent size effect was different along PC1 when accounting for groups (Figure 7a,b). PC1 coordinates were significantly and rather strongly correlated with the log‐transformed centroid sizes in bipedal archosauriforms (*r²*: 0.54, *p* < 0.01, Figure 7a) but not in quadrupedal ones (*r²*: 0.07, *p* > 0.1, Figure 7a). However, the two groups followed the same allometric trajectories within locomotor modes (Figure 7b), with similar correlations between PC2 coordinates and log‐transformed centroid sizes among bipedal archosauriforms (*r²*: 0.32, *p* < 0.01) and quadrupedal ones (*r*²: 0.19, *p* < 0.01, Figure 7b).

## 4 DISCUSSION

### 4.1 How do femoral shape changes correlate with locomotor habits and body size across the Triassic–Jurassic boundary?

#### 4.1.1 Covariation between locomotor modes and body size

An increase in femoral robusticity – increase of width relative to length – correlates with an increase in body size in terrestrial tetrapods (Biewener, 1989; Campione & Evans, 2012; Carrano, 1998; Etienne et al., 2020; Mallet et al., 2020), meaning that our results enabled us to study the shape variation linked to locomotor mode and size in early archosauriforms through early crown archosaurs (Figures 3a‐i, 4a,b). Moreover, our results demonstrate that the increase of femoral robusticity in early archosauriforms was coupled with the fourth trochanter being located closer to the mid‐shaft (i.e., more distally located) among the most robust femora. This is typically recognized as a signal of graviportality rather than cursoriality; i.e., a morphology favoring production of greater hip joint torques rather than larger ranges of femoral motion during retraction (Carrano, 1999; Coombs, 1978; Parrish, 1986). Therefore, our results highlight a covariation between the traits linked to locomotor mode and body size among early archosauriforms (Figures 3a‐i, 4a,b). Furthermore, our findings demonstrate that there was no restriction of locomotor mode depending on body size for Triassic–Jurassic archosauriforms, meaning that a bipedal habit (e.g., Table 2) was not restricted to small, more cursorial animals and that a quadrupedal habit was not exclusive to more graviportal ones (Figures 3a, 4a,b). However, the distinction between locomotor modes was clearer among robust femora than between gracile femora (Figure 3a). One of the main morphological differences between locomotor modes was femoral curvature (Figures 3g, 4b). The bipedal sauropodomorphs *Plateosaurus* and *Mussaurus* had an anteriorly bowed femur, whereas the quadrupedal aetosaurs *Typothorax* and *Paratypothorax* had a nearly straight one (Figure 3a; “Pla, Mus, Typ, Par,” 3G). Femoral curvature is negatively correlated with increased body size in dinosaurs (Carrano, 2001), and we demonstrated that this applies more broadly to archosauriforms. However, we found the opposite trend in our limited sample of crocodylian ontogeny (Figure 3a, 3g; see also Hedrick et al., 2021). Femoral curvature was enhanced plesiomorphically in avemetatarsalians with the origin of an erect limb posture and perhaps bipedalism (Hutchinson, 2001). This femoral curvature was retained by early bipedal sauropodomorphs and was subsequently lost by gigantic quadrupedal sauropods later during the Jurassic and Cretaceous (Carrano, 2001; Hutchinson, 2001). Thus, femoral curvature in our sample was mainly impacted by locomotor mode but also by body size, explaining why the distinction between bipedal and quadrupedal early archosauriforms was stronger among heavier animals than among lighter ones (Figure 3a). Nevertheless, this observation highlights an important morphological convergence in the specialization to graviportality—or at least to a greater body size—at the femoral level between bipedal avemetatarsalians and quadrupedal pseudosuchians (Figures 3a, 6). Our finding contributes to previous inferences from other skeletal elements (except for Kubo & Kubo, 2016) that avemetatarsalians were already morphologically disparate at the end of the Triassic and did not have a smaller body size in general than pseudosuchians (Brusatte et al., 2008a, 2008b; Foth et al., 2016, 2021; Kubo & Kubo, 2016; Stubbs et al., 2013; Toljagić & Butler, 2013).

#### 4.1.2 Similar femoral disparity between avemetatarsalians and pseudosuchians

Our dataset did not enable us to compare shifts of disparity across the Triassic–Jurassic transition because it mostly included archosauriforms from the Late Triassic. However, our results demonstrated that the femoral disparity of early avemetatarsalians was as high as that of pseudosuchians among the Late Triassic archosauriforms we sampled (Figures 3a, 6). Femoral robusticity ranged from the gracile morphology of the small lagerpetid *Kongonaphon*—similar to the most gracile pseudosuchians of our sample (the non‐crocodylian crocodylomorphs *Terrestrisuchus* and TTU‐P11443)—to the robust morphology of the heavy bipedal sauropodomorphs *Mussaurus* and *Plateosaurus*, similar to the most heavily‐built pseudosuchians *Crocodylus*, aetosaurs, *Revueltosaurus*, *Riojasuchus*, *Nundasuchus*, loricatan NMMNH P‐36144, suchian NMT RB187, and phytosaurs (Figure 3a; “Kon, Ter, Crm, Mus, Pla, Cro, Typ, Par, Rev, Rio, Nun, Lor, Suc, Phy,”). Additionally, both locomotor modes were represented in the two clades (Figures 3a, 6). Even if the locomotor mode was restricted to bipedal for the most robust avemetatarsalian femora and quadrupedal for the most robust pseudosuchian femora, our results showed that some avemetatarsalians—at least *Asilisaurus* and *Teleocrater*—also were assigned to a quadrupedal locomotor mode and some pseudosuchians—at least *Shuvosaurus*—were assigned to a bipedal locomotor mode (Figure 3a; “Asi, Tel, Shu,” 6; Table 2). Thus, the femoral disparity of early avemetatarsalians in comparison with pseudosuchians seems higher than previously suspected in other morphological studies that included femoral characters among other bones (Brusatte et al., 2008a, 2008b; Kubo & Kubo, 2012). Indeed, our finding could indicate that femoral disparity was underappreciated by studies which showed significant differences in disparity between the two clades in the Late Triassic by relying either on the whole skeleton (Brusatte et al., 2008a, 2008b) or ratios between limb element lengths (Kubo & Kubo, 2012). Furthermore, a substantial number of studies of the disparity of pseudosuchians and non‐archosaurian Archosauriformes around the Late Triassic and Early Jurassic were based on cranial characters. Hence these studies did not account for limb disparity in relative to locomotor habit and body size, which are often cited as central aspects in the faunal turnover across the Triassic–Jurassic boundary (Foth et al., 2016, 2021; Singh et al., 2021; Stubbs et al., 2013; Toljagić & Butler, 2013).

Brusatte et al. (2008a, 2008b) found that dinosaurs and ornithodirans as a whole had a lower disparity than pseudosuchians in the Late Triassic using a cladistic character dataset and a principal coordinate analysis including characters from the whole skeleton, whereas we found a similar level of disparity between these two clades when studying femoral shape variation using 3D GMM and PCA (Figures 3a, 6). Despite the inherent differences between our two approaches, here we have shown that avemetatarsalian (femoral) disparity could be enhanced by the inclusion of the early avemetatarsalians *Asilisaurus* and *Teleocrater*, as also shown by Toljagić and Butler (2013), who investigated pseudosuchian disparity using cranial characters. *Asilisaurus* and *Teleocrater* are early, possibly quadrupedal (Table 2) avemetatarsalians that were not known in 2008 (Nesbitt et al., 2011; 2017). Using the ratio between relative forelimb and hindlimb length and between metatarsal III and femur length, Kubo and Kubo (2012) measured greater morphological cursoriality among ornithodirans than pseudosuchians, mostly because the bipedal pseudosuchian *Poposaurus* had a lower “cursoriality index” than ornithodirans did. However, we found that *Poposaurus* showed a similar femoral robusticity (i.e., morphological cursoriality) to other cursorial avemetatarsalians (Figure 3a; “Pop,” 6). Moreover, we found that *Shuvosaurus*, another bipedal pseudosuchian, and *Terrestrisuchus*, a quadrupedal pseudosuchian, had a higher morphological cursoriality of the femur than *Poposaurus* (Figure 3a; “Shu, Ter, Pop”). The addition of *Shuvosaurus* and *Terrestrisuchus* could impact the findings of Kubo and Kubo (2012) on the relative length between the metatarsal III and femur since they were not included in their study. Nevertheless, our results indicate that the femoral morphology of *Poposaurus* and some specimens of *Shuvosaurus* was not as unambiguously bipedal as in avemetatarsalians. We inferred this result to most likely be caused by phylogenetic inertia, due to a combination of specialization to bipedalism and anatomical features specific to pseudosuchians, the vast majority of which in the Late Triassic were (plesiomorphically) quadrupedal animals. In addition, using metatarsal III and femur length, Kubo and Kubo (2012) indicated that sauropodomorphs were still more cursorial than large pseudosuchians, whereas our findings showed that larger avemetatarsalians and pseudosuchians, such as sauropodomorphs, *Riojasuchus*, aetosaurs, early diverging suchian NMT RB187 and loricatan NMMNH P‐36144 and *Nundasuchus*, had similar levels of femoral specialization to body size (i.e., femoral robusticity and fourth trochanter's position; Figure 3a; “Pla, Mus, Rio, Typ, Par, Suc, Lor, Nun,” 6). These findings are somewhat incongruent but may indicate that specialization to a heavy weight similarly impacted the femoral morphology—independently of femoral length—in each clade and that the 3D morphology of metatarsal III should be investigated further.

#### 4.1.3 Locomotor mode prediction based on femoral morphology and its evolutionary importance

Variations of femoral head rotation, shaft curvature, and fourth trochanter symmetry (as represented by PC2) in our sample were more driven by locomotor mode attribution than clade membership, even though the phylogenetic signal was significantly strong (Figures 3a,f‐i, 6; Table 2). This was highlighted by the quadrupedal avemetatarsalian *Teleocrater* lying close to pseudosuchians in the morphospace, much as a subset of bipedal pseudoschians *Shuvosaurus* and *Poposaurus* lay close to avemetatarsalians (Figure 3a “Tel, Shu, Pop,” 6; Table 2). Therefore, 3D femoral morphology appears useful for locomotor mode estimation, especially given that (1) 93.1% of specimens accompanied by *a priori* knowledge of locomotor modes were correctly estimated; (2) angles associated with femoral head rotation and distal condyles (i.e., crista tibiofibularis and lateral condyle) were both significantly associated with “known” and estimated locomotor modes (Figure 5a,b; Table 2). It is generally uncommon that both a fossilized hind‐ and forelimb are found preserved together in Late Triassic archosauriforms and in the vertebrate fossil record in general, sometimes with little evidence that they belonged to the same individual, which is problematic for estimations based on relative length between different segments from the appendicular skeleton. Therefore, our study adds to the understanding of locomotor mode predictions based on a single limb element and provides an alternative to estimations using femoral and/or humeral minimal circumference (McPhee et al., 2018).

Interestingly, both specimens of the silesaurid *Asilisaurus* were estimated as quadrupedal (Figure 3a; “Asi,” 6; Table 2). However, all other Silesauridae were estimated as bipedal (Figure 3a; “Sid, Sil,” 6; Table 2). This estimation is not congruent with the previously suggested locomotor mode of *Silesaurus*, which was described as a quadruped based on its limb proportions and trunk length (Fechner, 2009; Kubo & Kubo, 2012; Grinham et al., 2019; Table 2), although *Silesaurus* was originally described as a biped (Dzik, 2003). Piechowski and Dzik (2010) and Piechowski and Tałanda (2020) speculated that occasional bipedalism was possible for *Silesaurus* because its center of mass was presumed to be situated near its sacrum/hips, but this has never been quantified or compared with other bipeds/quadrupeds. Hence, the locomotor mode of Silesauridae remains uncertain even though our data bring new evidence for considering the controversial question of silesaurid locomotion, as well as suggesting that further analysis of locomotion of the clade using quantitative evidence based on other osteological elements than the femur alone may be warranted.

We made a similar observation with the lagerpetid *Dromomeron gregorii*, to which we initially assigned an ambiguous locomotor mode; same as its smaller relative *D*. *romerii* (Nesbitt et al., 2009). We inferred *D*. *gregorii* to be a quadruped except for one (out of three; the most mature juvenile specimen TMM 31100 1308; Figure 3a; “Dro”; Table 2). In contrast, Grinham et al. (2019) assumed that this *D*. *gregorii* was a facultative biped. *Postosuchus* and *Riojasuchus* also were estimated as either bipedal or quadrupedal in our analysis depending on the specimen (Table 2). *Riojasuchus* was described as possibly being a facultative biped given the prominent lesser trochanter on the femur, the shortened forelimb morphology and the relative lengths of digits between hind and forelimb in ornithosuchids (Walker, 1964; Baczko et al., 2020), which may explain why our estimation was different for each specimen. Bishop et al. (2020) obtained similar results for *Riojasuchus* using estimated mass properties and relative hindlimb and forelimb lengths, but noted the controversial nature of this taxon's locomotor habit, whereas Grinham et al. (2019) assumed *Riojasuchus* to be an obligate quadruped. However, *Postosuchus* was described, assumed, or estimated as an obligate biped in several recent studies (Bishop et al., 2020; Grinham et al., 2019; Weinbaum, 2013).

Considering that our study did not test for facultative bipedalism, this may highlight why some taxa were misclassified. Explanations other than facultative bipedalism include phylogenetic history, with only some clades retaining a plesiomorphically quadrupedal morphology for their femora whereas other skeletal elements indicate bipedalism. Hence, when estimations of locomotor mode based on femoral morphology only are ambiguous, estimated mass properties (Bishop et al., 2020) and other bones from both girdles (Grinham et al., 2019; Kubo & Kubo, 2012; McPhee et al., 2018) and the vertebral column (Bishop et al., 2020; Christian & Preuschoft, 1996; Jones et al., 2021; Padian, 2008) should be analyzed to better characterized locomotor habit of extinct archosauriforms.

Ontogenetic differences in locomotor mode did not seem to affect femoral morphology, as suggested by both adult and juvenile specimens of *Mussaurus* being estimated as bipedal in our study (Table 2), contrary to the hypotheses of Otero et al. (2019), Bishop et al. (2020), and Chapelle et al. (2020), who estimated juvenile *Mussaurus* as being quadrupedal and adults as bipedal using mass properties and limb bone relative lengths and circumferences. Similarly, it is not possible to demonstrate a shift in locomotor mode linked with growth (assessed via a limited cross‐sectional metapopulational sample) between the shortest and longest femora of *Postosuchus* and *Riojasuchus* (Tables 1, 3). However, we found that the juvenile specimens of *Mussaurus*, *Coelophysis*, and *Tawa* were located closer to the quadrupedal morphospace than the adult specimens (Figure S6; “Mus, Coe, Taw”). Similarly, the most mature individual of *Dromomeron* was closer to the bipedal morphospace than the quadrupedal one (Figure S6), leading to the most mature specimen of *Dromomeron* being estimated as bipedal, as discussed above in regard to facultative bipedalism (Table 2). Furthermore, we found the same ontogenetic spread along femoral specialization to locomotor mode in the extant crocodylian *Crocodylus*, with juvenile individuals laying closer to the bipedal morphospace than the adult one, while still being consistently estimated as quadrupedal (Figure S6; “Cro”). Hence, we infer this ontogenetic femoral disparity to be linked to a shift in how locomotor functional constraints were distributed across the appendicular skeleton toward adult stages, but not to a strict shift of locomotor mode across ontogeny. Nevertheless, our results indicate that those specimens should be investigated further using other approaches that can estimate shifts of locomotor mode and center of mass across ontogeny (e.g., Bishop et al., 2020; Otero et al., 2019), and ideally explain such shifts and locomotor function itself using fundamental biomechanical processes and mechanisms.

**FIGURE 8:**
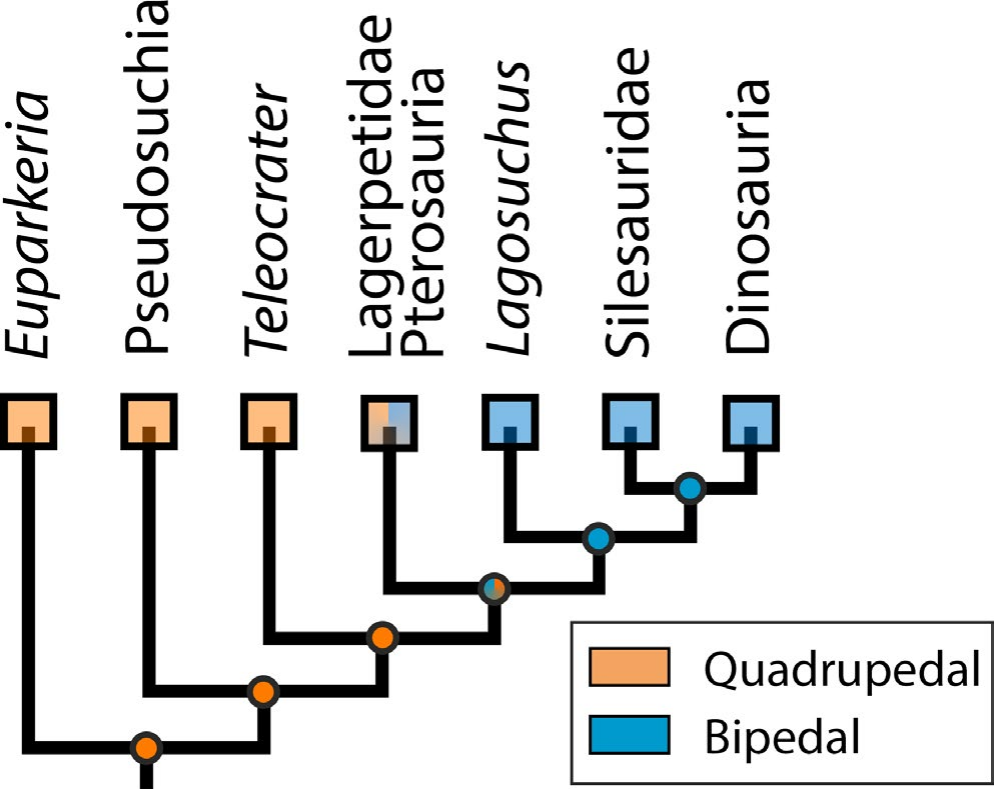
Evolutionary history of archosauriform locomotor modes under maximum parsimony assumption: colored in blue, bipedal; gradient, indeterminate; orange, quadrupedal. Squares represent character optimizations and circles are ancestral state reconstructions. Silesauridae shown as bipedal but see text for controversy over locomotor mode(s)

Regardless, our results raise an additional question prompted by available data and inferences: when did (obligate) bipedalism evolve in archosaur lineages? First, the estimation of locomotor mode regarding pterosaurs and lagerpetids, which were recently suggested to be sister taxa (Ezcurra et al., 2020), as well as silesaurids, is controversial (Padian, 1983, 2008; Grinham et al., 2019; Mazin et al., 2003; Mazin & Pouech, 2020; McCabe & Nesbitt, 2021; Piechowski & Dzik, 2010; Piechowski & Tałanda, 2020; Witton, 2015). Secondly, *Lagosuchus* clearly was bipedal like all early dinosaurs seem to have been (Bishop et al., 2020; Grinham et al., 2019), and Archosauria was ancestrally quadrupedal (Figure 1), with this plesiomorphic condition retained by *Teleocrater* among avemetatarsalians. Certainly all origin(s) of obligate bipedalism in Pseudosuchia were independent acquisitions (e.g., Bates & Schachner, 2012; Gauthier et al., 2011). Hence the above question can be reframed as, when did the dinosaur lineage first become bipedal? The ancestral locomotor mode on the avemetatarsalian lineage remains ambiguous (under maximum parsimony assumptions; see Figures 6, 8) until the Dinosauriformes (Dinosauria + Silesauridae + *Lagosuchus*) node. This ambiguity would be removed or reduced if some taxa with indeterminate locomotor modes were reassigned as facultative bipeds or if, as has been suggested, some Silesauridae independently reverted to quadrupedalism (see Grinham et al., 2019), which is, however, curiously contradicted by our findings for *Asilisaurus* being estimated as quadrupedal vs. *Silesaurus* being estimated as bipedal (Table 2). Our results suggest that a fresh look at the origin(s) of bipedalism within Avemetatarsalia is sorely needed through a combined approach including biomechanics, functional morphology, and phylogenetics (see also McCabe & Nesbitt, 2021).

### 4.2 Impact of locomotor mode and body size on features commonly used in cladistic analyses

We have shown that the 3D morphological variation of the femur linked to locomotor modes follows the inferred phylogeny, and that the variation linked to body size was strongly convergent between the avemetatarsalians and pseudosuchians (Figure 6). These observations enabled us to isolate which femoral characters and character states that are commonly used in archosaur phylogenetics might have homoplastic distributions corresponding to changes in body size, and also identify features that may vary more strongly with differences in locomotor mode (Figure 6).

The widening of the proximal end of the femur along the mediolateral axis was related to the variation of femoral robusticity, and influenced the medial and lateral sides of the epiphysis, but not the anteromedial and anterolateral tubers (Figures 3a‐e, 4a, 6). Consequently, the posteromedial tuber appeared larger than the anteromedial tuber on the more robust theoretical shape, which is a phylogenetic character usually attributed to most crocodylians, aetosaurs, *Revueltosaurus*, and ornithosuchids (Nesbitt, 2011; Novas, 1996; Figure 6). These two tubera are usually coded as equal in size in sauropodomorph dinosaurs, which shared a similar level of femoral robusticity as taxa mentioned above. Hence, we consider the variation of this phylogenetic character as homoplastic because it appeared convergently in pseudosuchians and avemetatarsalians (Figures 3a‐e, 4a, 6), which future studies should further analyze and consider. A similar observation is made on the medial edge of the fourth trochanter, which was rounded among robust femora and sharper on gracile ones (Figures 3a‐c, 4a); this anatomical variation, used in some cladistic analyses (e.g., Bennett, 1996; Nesbitt, 2011), also appears homoplastic at least within archosauromorphs.

However, the distal ridge of the fourth trochanter had a steep slope on bipedal femora and was more symmetric on quadrupedal femora (Figures 3g, 4b). A steeper slope of this distal ridge is characteristic of almost all Triassic dinosaur clades including *Herrerasaurus* but not most other theropods (Langer & Benton, 2006; Nesbitt, 2011). Accordingly, such theropods did not have a distal ridge as steep as that in bipedal sauropodomorphs, suggesting a continuous trait among saurischians (Figure 3a). This asymmetry in the fourth trochanter among dinosaurs was named “semi‐pendant” by Langer and Benton (2006) and interpreted as reflecting an increase in muscular stress on the distal part of the femur in early dinosaurs. A pendant fourth trochanter has long been assumed to correlate with the connection to a secondary tendon of the *M*. *caudofemoralis longus* (Dollo, 1888; Hutchinson, 2001). Its covariation with locomotor habits hints at a link with the origin of bipedalism and a more adducted, upright limb posture (Figures 3a,f‐g, 4b). Ornithischians displayed a more extreme state of this morphology, with a pendant fourth trochanter that has a reversed distal slope (Dollo, 1888; Hutchinson, 2001; Persons & Currie, 2020). However, because of the gap in the Triassic ornithischian fossil record (Irmis et al., 2007; Müller & Garcia, 2020), it is difficult to investigate the evolution of this feature and how it relates to the semi‐pendant state of saurischians alongside the origin(s) of bipedalism. Nevertheless, our observation that *Lesothosaurus*, a small ornithischian from the Early Jurassic, and the saurischians *Plateosaurus*, *Mussaurus*, and *Herrerasaurus* all had a similar slope of the distal ridge of the fourth trochanter, despite having pendant to semi‐pendant morphologies, respectively might illuminate the evolutionary history of the fourth trochanter among dinosaurs and how the reversed‐distal slope of this muscular attachment appeared. However, this character state could also be plesiomorphic because of the ancestral diapsid presence of the secondary “tendon of Sutton” (Dollo, 1888; Hutchinson, 2001). Nevertheless, our finding that the fourth trochanter might have at least two distinct components of morphological variation that are often coded in phylogenetic analyses, with the medial ridge being homologous and the distal slope homoplastic, could inspire follow‐up research, including phylogenetic analyses (Figures 4b, 6).

The long axis of the femoral head was plesiomorphically more anteriorly oriented in pseudosuchians and more medially oriented in avemetatarsalians (Figures 4b, 6). This feature is known to distinguish the two clades without indicating a bipedal/quadrupedal locomotor mode, because quadrupedal dinosaurs that evolved after the Triassic–Jurassic transition did not return to the ancestral condition of an anteriorly oriented femoral head (Carrano, 2000; Hutchinson, 2001). However, we suggest that the functional significance of femoral head orientation may be underappreciated (Figures 3a,h‐i, 4b, 5a). A commonly suggested functional explanation of this feature is that the anteriorly oriented femoral head correlates with a (plesiomorphically) more sprawled hindlimb posture and rotary gait whereas a medially oriented femoral head evolved in lineages having a more erect (adducted) limb posture and parasagittal gait (Bonaparte, 1984; Carrano, 2000; Charig, 1972; Demuth et al., 2020; Hutchinson, 2001, 2006). Our study did not address the difference of postures between sprawling to erect, and some archosauriforms were not “fully erect.” Thus, we considered a continuum in postures between sprawling to erect (e.g., see Gatesy, 1991 and Hutchinson, 2006) and a more erect limb posture as a prerequisite to bipedalism in both archosaur clades. In addition, the variation of femoral head orientation demonstrated that the bipedal pseudosuchian *Shuvosaurus* and the potentially bipedal pseudosuchian *Postosuchus* have a more medially oriented femoral head than other pseudosuchians, which were quadrupedal (or controversially so) (Figure 3a; “Shu, Pop,” Figures 3h‐i, 4b, 5a, 6; Tables 2, S3). This was not the case for the bipedal pseudosuchian *Poposaurus* (Figure 3a; “Pop,” Figure 6). However, both *Poposaurus* specimens were close to the “least” bipedal femur of *Shuvosaurus*. We made the same observation with the quadrupedal (or potentially so) avemetatarsalians *Teleocrater*, *Asilisaurus*, and *Dromomeron gregorii* having a more anteriorly oriented femoral head than clearly bipedal avemetatarsalians (Figure 3a; “Tel, Asi, Dro,” Figures 4b, 6; Tables 2, S3). Thus, femoral head orientation could be even more closely related to locomotor mode and kinematics than previously thought. Analyses of joint mobility (e.g., Demuth et al., 2020) could test this possibility further.

We infer that the lesser trochanter is less important than femoral head orientation or bone curvature in the estimation of early archosaur locomotor modes. We showed that the lesser trochanter was more expanded proximo‐anteriorly among bipedal archosaurs and most avemetatarsalians (Figures 3g‐h, 4b, 6). A well‐developed lesser trochanter evolved independently in different clades of dinosaurs and has been suggested to correlate with bipedalism, as it could allow a greater protraction and retraction of the hindlimb (Carrano, 2000; Gauthier, 1986; Novas, 1996). However, a lesser trochanter is absent in the bipedal pseudosuchians *Shuvosaurus* and *Poposaurus* (Nesbitt, 2007; Schachner et al., 2019), supporting the inference that this feature appeared only in bipedal avemetatarsalians, with parasagittal gait as a prerequisite (Carrano, 2000). Moreover, one specimen of *Riojasuchus*, perhaps a facultatively bipedal ornithosuchid (Baczko et al., 2020), resembled more quadrupedal pseudosuchians whereas the other specimen was closer to bipedal archosaurs, such as members of Poposauridae, in the morphospace, despite having a proximo‐anteriorly developed lesser trochanter (Figure 3a; “Rio, Pop”).

An anteriorly bowed (curved) femur is also a character used to distinguish the in‐group relationships within archosaurs (Figures 3a, 4b, 6; Sereno, 1991), varying according to locomotor habit (Figure 3g) and we infer this feature to be a reliable predictor of locomotor mode. Femoral bowing is known to vary across all archosauriforms (Gauthier et al., 1988, more specifically with the origin of a more erect posture (Hutchinson, 2001) and body size variations (Biewener, 1983; Carrano, 2000). This feature is suggested to better predict mechanical bending stress related to a bipedal locomotor habit (Hutchinson, 2001), whereas a straightening of the shaft correlates with increased body mass in quadrupedal animals, except across crocodylian ontogeny (Biewener, 1983; Carrano, 2000, Hedrick et al., 2021; our results for *Crocodylus*). However, large bipedal archosaurs, such as sauropodomorphs and theropods, retained an anteroposteriorly bowed femur (Hutchinson, 2001). Thus, the variation of this feature has a strong functional implication and might be well suited to predict archosaur posture and locomotor mode, especially because bipedal pseudosuchians are thought to have had a more erect hindlimb posture similar to that of bipedal ornithodirans (Figures 3a, 4b, 6; Bates & Schachner, 2012; Nesbitt & Norell, 2006).

The angle between the lateral condyle and the crista tibiofibularis (Figure 2a) is known to distinguish ornithosuchids, aetosaurs, *Revueltosaurus*, phytosaurs and most avemetatarsalians with a rather obtuse angle from other archosauriforms such as *Postosuchus*, poposauroids, and crocodylomorphs, with a rather right angle (Nesbitt, 2011; Parker & Irmis, 2005; Parrish, 1986). However, our study demonstrates that, despite varying continuously rather than in a discrete manner, this angle is greater (more obtuse) in quadrupedal archosauriforms (most pseudosuchians) than in bipedal ones (most avemetatarsalians, Figures 3a, 4b, 5b; Table S3). Moreover, the phylogenetic signal of the variation between locomotor modes shows that the mean angle of this feature is significantly greater (more obtuse) in pseudosuchians than in avemetatarsalians (more acute), with exceptions for some taxa (Figures 3a, 5b, 6); contradicting prior ideas that avemetatarsalians, some pseudosuchians and phytosaurs shared the same angle (Nesbitt, 2011; Parker & Irmis, 2005; Parrish, 1986). Furthermore, the acuteness of this angle may be increased by the presence of a groove between the crista tibiofibularis and the lateral condyle among bipedal (or controversially so) poposauroids, dinosauromorphs, and *Postosuchus*, whereas this groove is absent in quadrupedal (or controversially so) phytosaurs, *Euparkeria*, aetosaurs, *Revueltosaurus* and *Riojasuchus* (Nesbitt, 2011). One possible explanation would be that the preservation of this groove may vary between specimens, subsequently affecting the acuteness of the angle, especially because of its proximity to the cartilaginous epiphyseal cap, which is not always well preserved in extinct archosauriforms (Bonnan et al., 2010; Holliday et al., 2010; Nesbitt, 2011). We did not observe a variation of the proximodistal width of bone epiphyses, at least not along the two first PC axes (Figures 3a‐i, 4), meaning that the variation in the amount of preserved distal articular cartilage does not directly explain the morphological variation shown in our study, even if it may be visible among other PC axes. Furthermore, the potential link between the acuteness of the angle and the presence of a groove between the crista tibiofibularis and the lateral condyle raise the issue of correlation between these two characters in phylogenetic analyses. Thus, despite the potential impact of taphonomic factors, the angle between the lateral condyle and the crista tibiofibularis should be investigated further in order to better understand its evolutionary history and functional implications such as knee joint mobility and orientation and should be better integrated into phylogenetic studies.

### 4.3 Size effect and crocodylian ontogeny

We found that an evolutionary allometric relationship of increasing femoral robusticity and centroid size was significant, but weak. This effect was due to bipeds and quadrupeds having different allometric trajectories involving an increase of femoral robusticity (Figures 3a‐e, 7a). This effect was already described before and is known to intensify with the phylogenetic breadth of a sample (Adams et al., 2013; Klingenberg & Froese, 1991; Mitteroecker et al., 2004). We show that, when accounting for groups (bipedal/quadrupedal), the correlation between the increase of femoral robusticity and centroid size was significant and strong among bipedal archosauriforms, but not among quadrupedal archosauriforms. Centroid sizes among the most robust quadrupedal femora were lower than among the most robust bipedal ones, with more scattered values along the morphological variation, whereas values were similar for the most gracile bones among the two groups (Figure 7a). We showed that this pattern does not result from the presence of juvenile crocodylians in the sample, because *Revueltosaurus* and *Parringtonia* also had low centroid sizes with highly robust femora (Figure 3a “Cro, Rev, Par,” Figure 7a, Figure S6). In addition, Dodson (1975) and Hedrick et al. (2021) demonstrated that the femoral robusticity in *Alligator mississippiensis* varied significantly along ontogeny, with the fourth trochanter migrating down the shaft toward the adult age, along with an increase of femoral disparity. This morphological variation is identical to the one we highlighted along the specialization to body size. Yet, we did not observe a separation between juveniles and the adult specimen of *Crocodylus* along the increase of femoral robusticity (as in PC1; Figure 3a; Figure S6; “Cro”), subsequently indicating the rather conservatively high robusticity in crocodile femora across ontogeny when compared with a larger taxonomic sample. However, we did observe a separation between juveniles and adult *Crocodylus* along the axes pertaining to femoral specialization to locomotor mode (as in PC2; Figure 3a; “Cro”). Thus, juvenile *Crocodylus* had straighter femora (i.e., lower anterior curvature) than the adult specimen, which is congruent with findings described under “femoral robusticity” in *Alligator* by Hedrick et al. (2021). Morphological variation of the femur that seemed to indicate a shift of estimated locomotor mode from bipedal to quadrupedal across ontogeny was also observed in extant crocodylians by McPhee et al. (2018; *Caiman*) and Bishop et al. (2020; *Alligator*). There is no known bipedalism in Crocodylia, even early in posthatching ontogeny, so these results are all anomalous in terms of identifying locomotor mode. The main difference we have highlighted with previous studies is that femora of juvenile *Crocodylus* showed a higher degree of specialization to a quadrupedal locomotor mode than adults (Figure 3a; Figure S6; “Cro”). This opposite trend in results could be explained by findings from Ijima and Kubo (2019) who recently discussed that growth parameters and variation of limb morphology along ontogeny were highly variable across the various extant clades of crocodylians, indicating that a trend observed in *Crocodylus* ontogeny may not be necessarily attributable to *Alligator* nor *Caiman*.

### 4.4 Convergence between semi‐aquatic lifestyle and specialization to heavy weight

Our results showed that phytosaurs and the extant pseudosuchian *Crocodylus* shared a similar femoral morphology both in term of robusticity and specialization to locomotor habit (Figure 3a; “Cro,” Figure 6; Table 2). This morphological convergence was already described at the level of whole‐animal morphology and suggested as an adaptation to a similar semi‐aquatic lifestyle (Lautenschlager & Butler, 2016; Stocker & Butler, 2013). However, we showed that other taxa with robust femoral morphology and a probable terrestrial lifestyle, such as aetosaurs, *Revueltosaurus*, ornithosuchids, and non‐sauropod sauropodomorphs, were recovered close to *Crocodylus* and phytosaurs in the morphospace, highlighting a morphological convergence between adaptations to weight support and a secondary semi‐aquatic lifestyle, with similarly enlarged epiphyseal width and a rounded fourth trochanter near the mid‐shaft; independently of locomotor habit (Figure 3a‐e; “Typ, Par, Rev, Rio, Pla, Mus,” Figure 4a). Such morphological convergence between heavy terrestrial and semi‐aquatic quadrupeds is intriguing because it evolved to serve opposite functions in relation to environmental factors (e.g., buoyancy in a low gravity environment vs. improved resistance to gravitational forces on land) and has already been studied among some massive animals through a microanatomical approach (Houssaye et al., 2016, 2021). We suggest that this convergence should be investigated further in archosauriforms using a similar approach coupled with biomechanical analyses in order to decipher specializations between these two functional constraints which seemed to drive the appearance of convergent femoral morphologies.

## 5 CONCLUSION

Our study demonstrates the link between femoral morphology and locomotor habits among early archosauriforms. First, we demonstrate that features such as femoral head orientation, distal slope of the fourth trochanter, femoral curvature, and the angle between the crista tibiofibularis and lateral condyle appear linked to locomotor mode. Conversely, the expansion of the lesser trochanter does not seem to indicate locomotor habits as clearly as it was previously suggested to do for early archosauriforms. Our work highlights that the shape signal associated with locomotor mode is stronger than the phylogenetic one, thereby providing reliable indicators to predict locomotor mode based only on femoral morphology (success rate of 93%), without relying on ratios of hindlimb and forelimb linear dimensions. Moreover, 3D GMM also provides information about morphological variation linked with locomotor mode estimations based on linear measurements (i.e., bone circumference and ratio between limb lengths) to clarify the reliability of estimations and detect complex interactions between traits, such as trade‐offs with specializations to body size. Secondly, we show that the fourth trochanter position and roundness as well as the widening of both epiphyses vary in conjunction with femoral robusticity, and together are linked to an increase in body size, thereby potentially constituting a suite of more or less graviportal specializations. Thirdly, we illuminate how deeply embedded locomotor habits and size increases are within the evolutionary history of archosauriforms. We also raise concerns that because the locomotor modes of some early archosauriforms, especially lagerpetids and silesaurids, remain ambiguous, the evolutionary polarity of quadrupedalism/bipedalism within Avemetatarsalia should be re‐investigated more mechanistically. Finally, we demonstrate that femoral curvature co‐varies between locomotor modes and cursoriality/graviportality, with a clear distinction between a straight and a curved shaft among robust femora. Furthermore, our study shows a decoupling in fourth trochanter shape variation that is associated with locomotor modes (symmetrical to semi‐pendant) and body size (sharp to rounded) as well as different allometric trajectories between bipedal and quadrupedal archosauriform femora. These examples of co‐variation, differences of trajectories, and decoupling emphasize the considerable amounts of convergent specialization to weight support in the femora of archosauriforms. This convergence in 3D femoral morphology is a cautionary note on the potentially high amount of homoplastic features and the necessity of accounting for body size when studying the evolutionary history of these animals. Nevertheless, our findings about the functional morphology of the femur in archosauriforms add to the understanding of the early evolution of dinosaurs and other archosauriforms during the faunal turnover that occurred across the Triassic–Jurassic transition.

## AUTHOR CONTRIBUTIONS

R. P. designed the experiments, digitized specimens, performed data analyses and interpretation, wrote the manuscript and approved the final draft. A. H. designed the experiments, participated in data interpretation, corrected the manuscript and approved the final draft. S. J. N. digitized specimens, participated in data interpretation, corrected the manuscript and approved the final draft. J. R. H. designed the experiments, digitized specimens, participated in data interpretation, corrected the manuscript and approved the final draft.

## Supporting information

Fig S1

Fig S2

Fig S3

Fig S4

Fig S5

Fig S6

Table S1‐S3
