# Supplementary figures and images for: Femoral specializations to locomotor habits in early archosauriforms
Source: J Anat. 2021 Nov 28;240(5):867–92. doi: 10.1111/joa.13598 (PMC9005686; doi:10.1111/joa.13598)

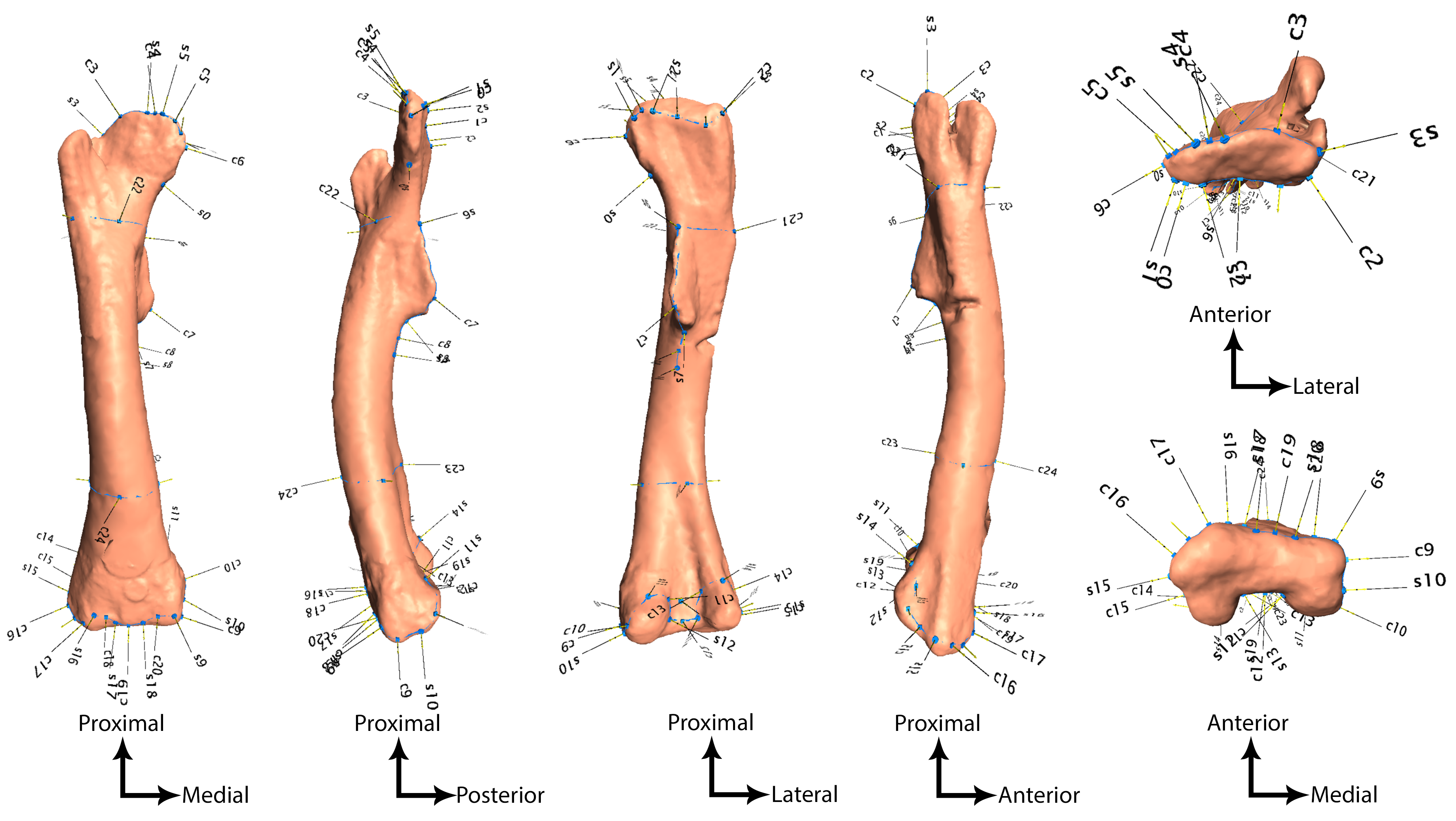

Supplement: Supplementary file 1 — Fig S1 [file JOA-240-867-s005.tif]

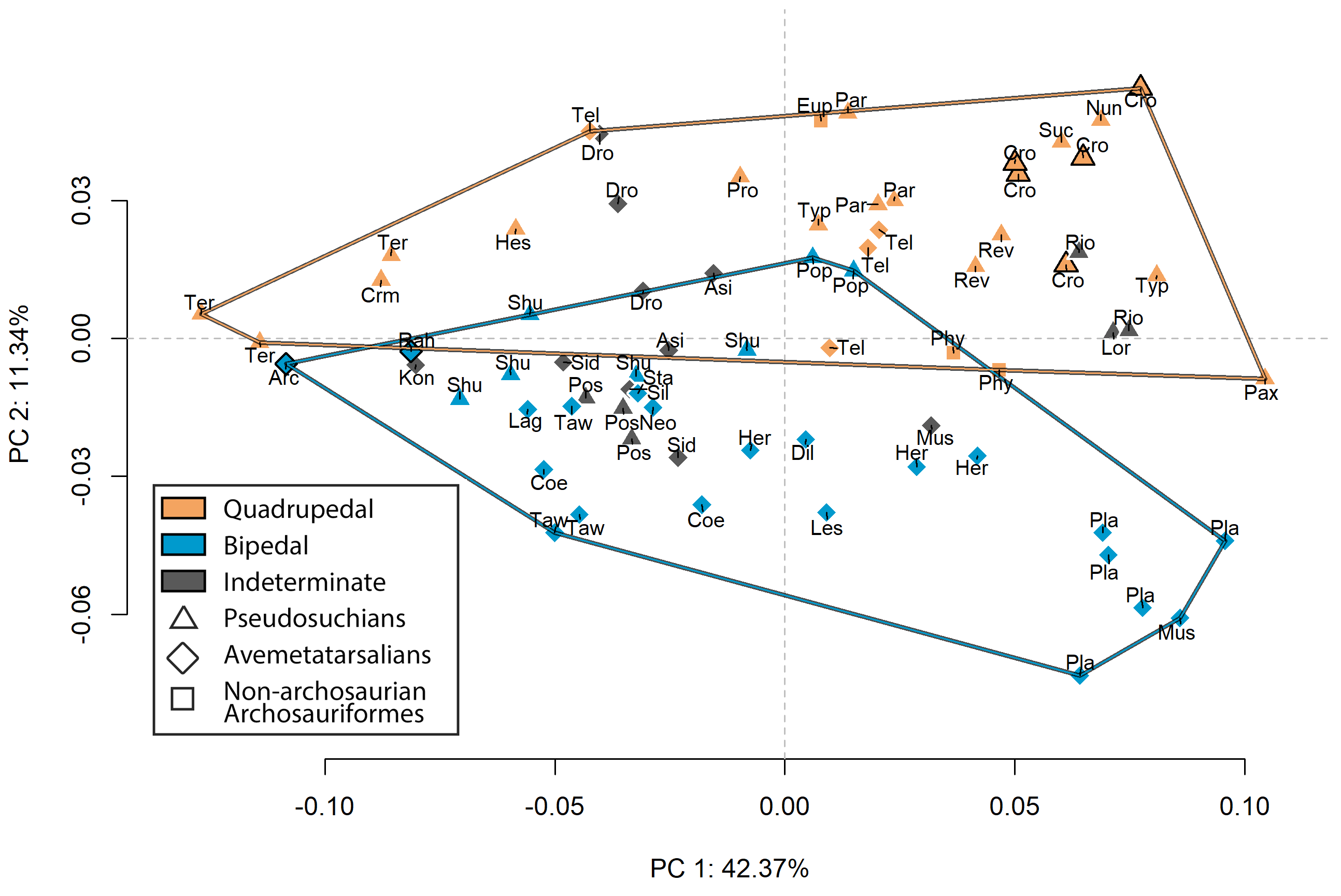

Supplement: Supplementary file 2 — Fig S2 [file JOA-240-867-s003.tif]

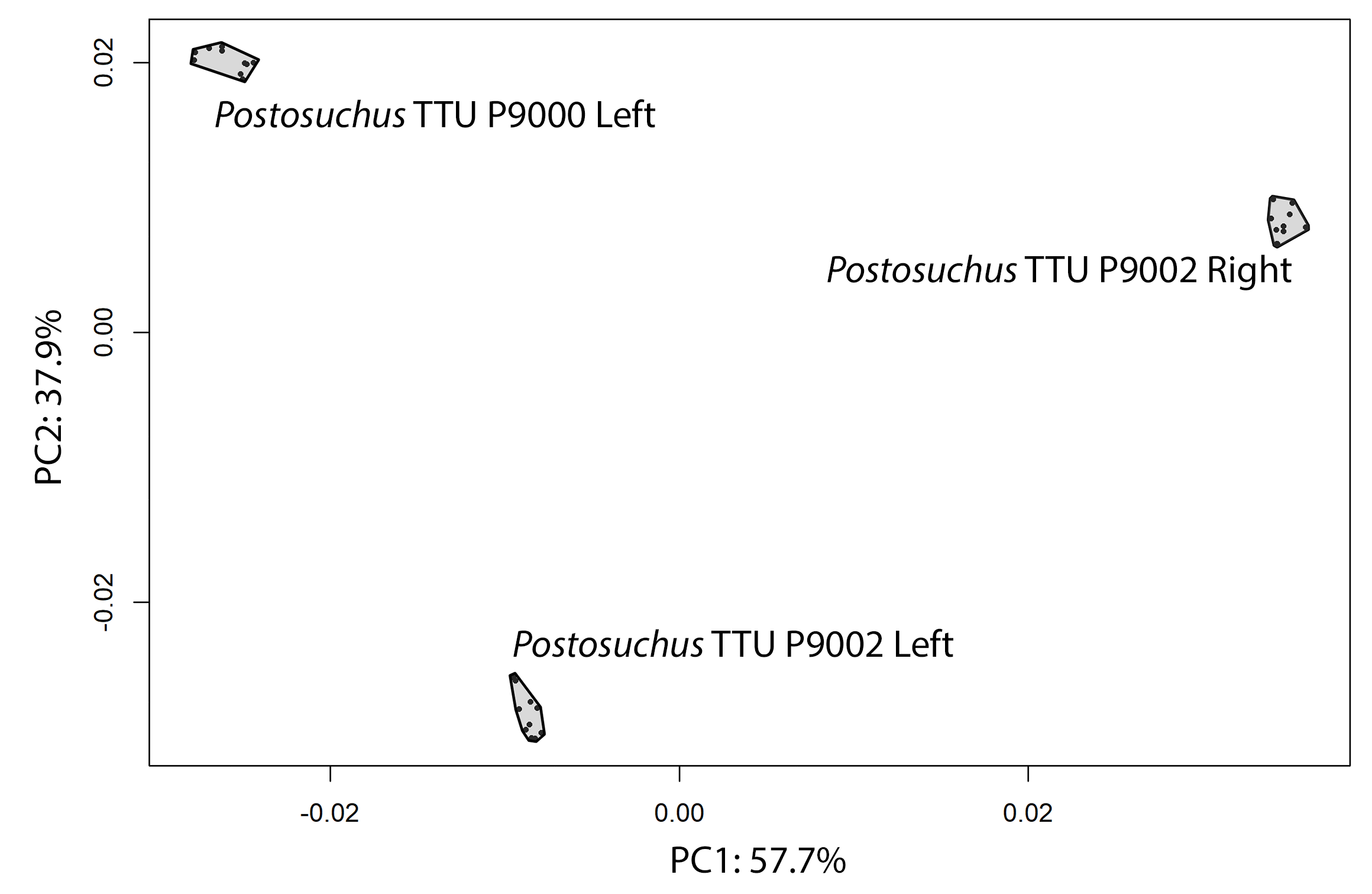

Supplement: Supplementary file 3 — Fig S3 [file JOA-240-867-s004.tif]

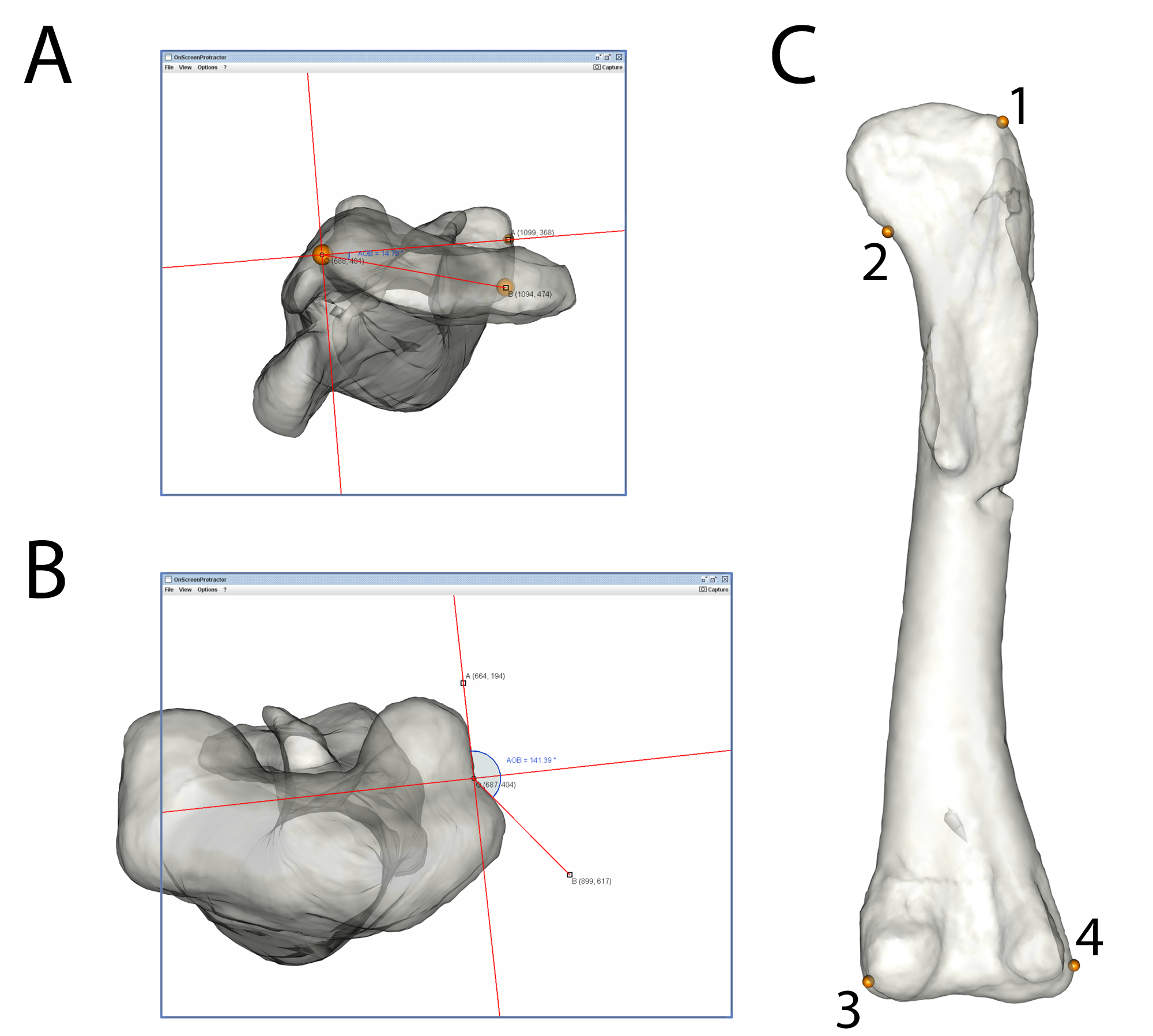

Supplement: Supplementary file 4 — Fig S4 [file JOA-240-867-s007.tif]

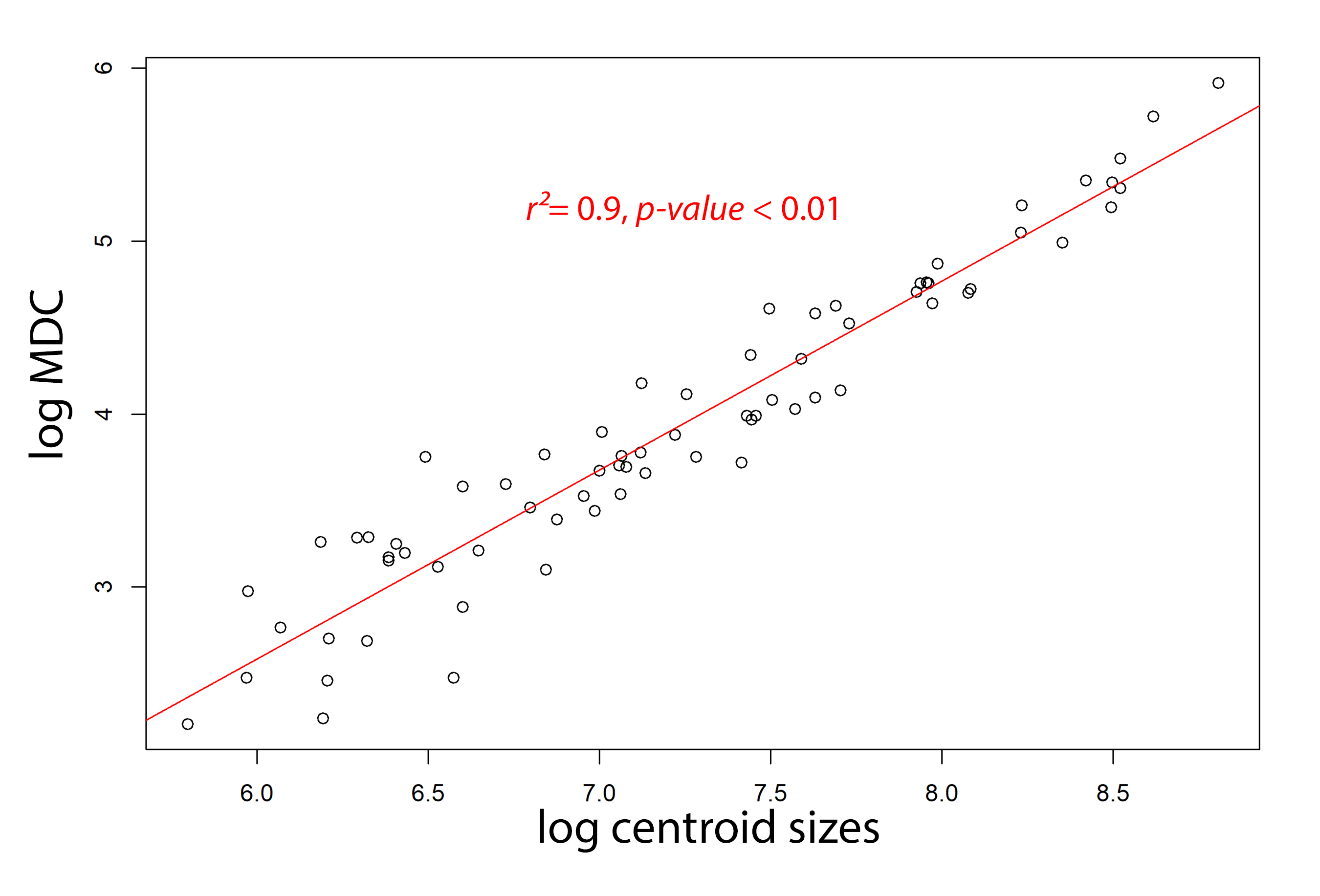

Supplement: Supplementary file 5 — Fig S5 [file JOA-240-867-s006.tif]

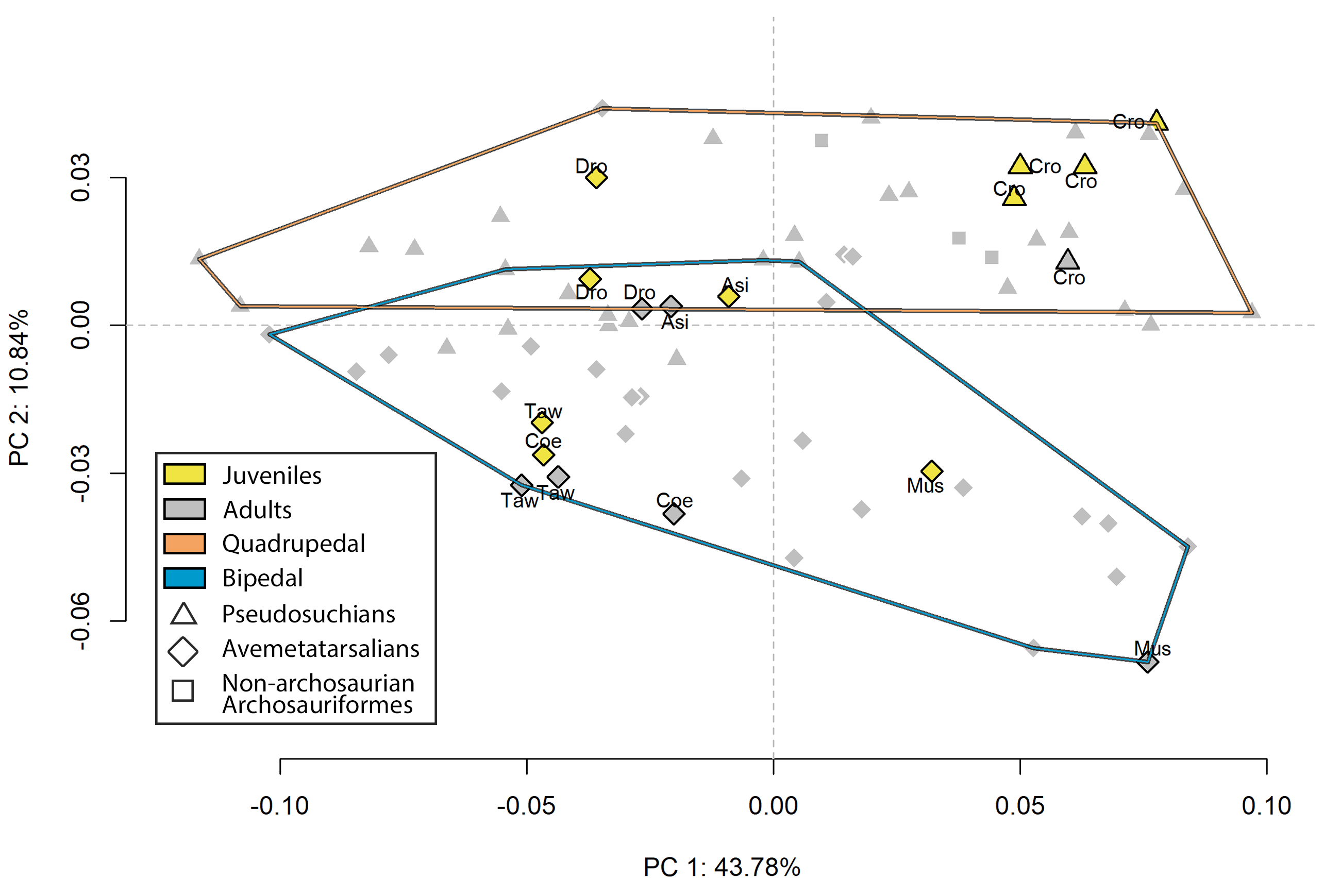

Supplement: Supplementary file 6 — Fig S6 [file JOA-240-867-s002.tif]
