# Supplementary material for: Femoral specializations to locomotor habits in early archosauriforms
Source: J Anat. 2021 Nov 28;240(5):867–92. doi: 10.1111/joa.13598 (PMC9005686; doi:10.1111/joa.13598)
Supplement: Supplementary file 7 — Table S1‐S3 [file JOA-240-867-s001.docx]

Table S1: CT and µCT scan parameters. Col. Nb. is institution and specimen number of specimen. CT, CT scan; µCT, micro-CT scan. Unlisted specimens or entries do not have available data.

| Taxa | Col. Nb. | Scan | Voltage (kV) | Current (µA) | Voxel size (µm) | Exposure duration (ms) | | Number of slices | Resolution (pixels) |
| --- | --- | --- | --- | --- | --- | --- | --- | --- | --- |
| *Euparkeria capensis* | SAM PK 5867 | µCT | 170 | 400 | 90 | 500 | 1792 | | 1387 x 515 |
| *Poposaurus gracilis* | YPM 57100 | CT | 140 |  |  | 500 | 164 | |  |
| *Terrestrisuchus gracilis* | NHMUK PV R7562 | µCT | 190 | 200 | 60 | 708 | 2661 | | 2000 x 2000 |
| *Terrestrisuchus gracilis* | NHMUK PV R10002 | µCT | 190 | 200 | 60 | 708 | 2048 | | 2000 x 2000 |
| *Terrestrisuchus gracilis* | Composite | µCT | 190 | 200 | 60 | 708 | NA | | 2000 x 2000 |
| *Protosuchus richardsoni* | AMNH 3024 | CT | 140 | 300 | 390 |  |  | |  |
| *Crocodylus niloticus* | DDNC01 | CT | 120 | 100 | 410 |  | 1769 | |  |
| *Crocodylus niloticus* | DDNC02 | CT | 120 | 100 | 410 |  | 1168 | |  |
| *Crocodylus niloticus* | DDNC03 | CT | 120 | 100 | 410 |  | 1434 | |  |
| *Crocodylus niloticus* | DDNC04 | CT | 120 | 100 | 410 |  | 1586 | |  |
| *Lagosuchus lilloensis* | PVL 4670 | CT | 120 | 61 | 70 | 250 | 1381 | | 363 x 592 |
| *Mussaurus patagonicus* | MPM 1813 | µCT | 150 | 815 | 109 |  |  | |  |
| *Plateosaurus* | GPIT RE7288 | CT |  |  | 500 |  |  | |  |
| *Dilophosaurus wetherilli* | UCMP 37302 | CT | 140 | 80 |  |  | 189 | |  |
| *Rahonavis ostromi* | UA 8656 | µCT | 130 | 110 | 100 | 200 | 924 | |  |

Table S2: Landmark scheme according to the numerotation shown in Figure S1. Abbreviations: s, anatomical landmarks; c, sliding semi-landmarks on curves.

| **N Land.** | **Description** |
| --- | --- |
| s0 | Maximum of concavity in the inflexion under the femoral head |
| s1 | Maximum of concavity in the posterior depression between the femoral head, *facies articularis antitrochanterica* and the anteromedial tuber |
| s2 | Maximum of concavity on the lateral ridge of the anteromedial tuber |
| s3 | Most proximal point of the lateral border of the greater trochanter |
| s4 | Maximum of concavity in the anterior depression between the femoral head, *facies articularis antitrochanterica* and the anterolateral tuber |
| s5 | Most anterior point of the anterolateral tuberosity |
| s6 | Most proximal point of the 4^th^ trochanter |
| s7 | Maximum of concavity of the distal part of the 4^th^ trochanter |
| s8 | Most distal point of the 4^th^ trochanter |
| s9 | Most anterior point of the medial condyle |
| s10 | Most medio-distal point of the medial condyle |
| s11 | Maximum of concavity in the proximal depression on the posterior end of the medial condyle |
| s12 | Maximum of concavity of the medial side of the posterior intercondylar fossa |
| s13 | Maximum of concavity of the lateral side of the posterior intercondylar fossa |
| s14 | Maximum of concavity in the proximal depression on the posterior end of the crista tiobiofibularis |
| s15 | Most latero-distal point of the lateral condyle |
| s16 | Most anterior point of the lateral condyle |
| s17 | Maximum of concavity of the lateral side of the anterior intercondylar fossa |
| s18 | Maximum of concavity of the medial side of the posterior intercondylar fossa |
| s19 | Maximum of concavity in the posterior intercondylar depression |
| c0 ; c6 | Delimitation of the articular surface of the proximal epiphysis |
| c7 ; c8 | Border of the 4^th^ trochanter |
| c9 ; c20 | Delimitation of the articular surface of the distal epiphysis |
| c21 ; c22 | Delimitation of the proximal region based on the most proximal point of the 4^th^ trochanter |
| c23 ; c24 | Delimitation of the distal region based on the point of abrupt change in the shaft circumference |

Table S3: Measured angle in degrees for the femoral head rotation (FH), between crista tibiofibularis (Cfb) and lateral condyle (Lc) and the minimum diaphyseal circumference of the femur (MDC).

| **Name** | **FH** | **Cfb-Lc** | **MDC (mm)** |
| --- | --- | --- | --- |
| *Archaeopteryx* HMN 1880 (R) | 7 | 153 | 11.7 |
| *Asilisaurus* NMT RB159 (L) | 89 | 171 | 40.6 |
| *Asilisaurus* NMT RB169 (L) | 48 | 164 | 25.8 |
| *Coelophysis* AMNH FARB 32843 (R) | 45 | 115 | 39.4 |
| *Coelophysis* UCMP129618 (R) | 9 | 155 | 62.7 |
| *Crocodylus* DDNC01 (R) | 63 | 164 | 26.7 |
| *Crocodylus* DDNC02 (R) | 48 | 165 | 19.6 |
| *Crocodylus* DDNC03 (R) | 40 | 159 | 26.1 |
| *Crocodylus* DDNC04 (R) | 53 | 160 | 26.8 |
| *Crocodylus* FNC5 (L) | 39 | 148 | 102.1 |
| *Dilophosaurus* UCMP37302 (L) | 33 | 92 | 180.3 |
| *Dromomeron* TMM31100 1308 (R) | 28 | 122 | 22.6 |
| *Dromomeron* TMM31100 464 (R) | 46 | 97 | 24.8 |
| *Dromomeron* TMM31100 764 (R) | 44 | 110 | 14.9 |
| *Euparkeria* SAMPK5867 (R) | 51 | 135 | 15.9 |
| *Herrerasaurus* MACN18060 (L) | 65 | 153 | 92.2 |
| *Herrerasaurus* PVL2566 (R) | 50 | 144 | 155.9 |
| *Herrerasaurus* PVSJ373 (L) | 26 | 129 | 103.5 |
| *Hesperosuchus* AMNH FR6758 (L) | 49 | 163 | 34.4 |
| *Kongonaphon* UA10618 (R) | 17 | NA | 9.1 |
| *Lesothosaurus* NHMUK RUB17 (R) | 15 | 141 | 36.4 |
| *Lagosuchus* PVL4670 (R) | 60 | 97 | 11.9 |
| *Mussaurus* MLP60-III-20-22 (R) | 2 | 127 | 370.2 |
| *Mussaurus* MPM 1813 (R) | 13 | 150 | 43.3 |
| Neotheropoda GR1046 (R) | 50 | 120 | 59.3 |
| *Nundasuchus* NMT RB48 (R) | 30 | 145 | 100.5 |
| *Paratypothorax* TTU-P12547 (R) | 35 | 138 | 182.3 |
| *Parringtonia* NMT RB188 (R) | 42 | 172 | 24.5 |
| *Parringtonia* NMT RB426 (L) | 29 | 144 | 23.9 |
| *Parringtonia* NMT RB426 (R) | 56 | 162 | 23.4 |
| Phytosauridae PEFO23347 (L) | 50 | 159 | 116.9 |
| Phytosauridae PEFO 31219 (L) | 54 | 162 | 116.4 |
| *Plateosaurus* GPIT RE7288 (R) | 24 | 110 | 210.9 |
| *Plateosaurus* SMNS13200a+e (L) | 27 | 117 | 305.1 |
| *Plateosaurus* SMNS91297 (L) | 19 | 141 | 239.6 |
| *Plateosaurus* SMNS91300 (L) | 3 | 97 | 201.4 |
| *Plateosaurus* SMNS91310 (L) | 25 | 134 | 208.2 |
| *Poposaurus* YPM 57100 (L) | 49 | 107 | 116.4 |
| *Poposaurus* YPM 57100 (R) | 58 | 83 | 110.6 |
| *Postosuchus* TTU-P9000 (L) | 45 | 103 | 147 |
| *Postosuchus* TTU-P9002 (L) | 62 | 104 | 110.2 |
| *Postosuchus* TTU-P9002 (R) | 37 | 105 | 112.5 |
| *Protosuchus* AMNH3024 (R) | 49 | 140 | 31.8 |
| *Rahonavis* UA8656 (R) | 16 | 162 | 17.9 |
| Loricata NMMNH P-36144 (L) | 21 | 118 | 130.3 |
| *Revueltosaurus* PEFO34269 (R) | 47 | 153 | 42.6 |
| *Revueltosaurus* PEFO34561 (L) | 32 | 139 | 35.9 |
| *Riojasuchus* PVL3827 (L) | 34 | 145 | 65.2 |
| *Riojasuchus* PVL3828 (L) | 67 | 172 | 61.2 |
| *Shuvosaurus* NMMNHP-4695 (L) | 55 | 119 | 31.2 |
| *Shuvosaurus* TTU-P18307 (R) | 46 | 130 | 54.1 |
| *Shuvosaurus* TTU-P18321 (L) | 39 | 113 | 52.9 |
| *Shuvosaurus* TTU-P18336 (L) | 44 | 131 | 60.1 |
| *Shuvosaurus* TTU-P9001 (L) | 53 | 142 | 56.2 |
| Silesaurid TMM31100 1303 (L) | 46 | 139 | 38.8 |
| Silesaurid TMM31100 185 (L) | 17 | 139 | 40.2 |
| *Silesaurus* ZPAL361.23 (L) | 47 | 127 | 41.3 |
| Sphenosuchian TTU-P11443 (R) | 56 | 147 | 22.2 |
| *Staurikosaurus* MCZ1699 (R) | 41 | 163 | 75.1 |
| Suchian NMT RB187 (R) | 61 | 148 | 49.2 |
| *Tawa* GR1033 (R) | 41 | 153 | 42.6 |
| *Tawa* GR1054 (L) | 20 | 146 | 54.1 |
| *Tawa* GR244 (L) | 39 | 150 | 29.7 |
| *Teleocrater* NHMUK PV R6795 (R) | 44 | 167 | 48.4 |
| *Teleocrater* NMT RB843 (R) | 44 | 154 | 43.7 |
| *Teleocrater* NMT RB844 (R) | 34 | 168 | 42.9 |
| *Teleocrater* NMT RB845 (R) | 63 | 149 | 34 |
| *Terrestrisuchus* 721.3 (R) | 45 | 99 | 11.9 |
| *Terrestrisuchus* R10002 (R) | 32 | 157 | 14.7 |
| *Terrestrisuchus* Composite (R) | 46 | 155 | 9.4 |
| *Typothorax* NMMNH-P11775 (L) | 45 | 151 | 76.8 |
| *Typothorax* NMMNH-P11778 (L) | 53 | 138 | 97.7 |
